# Structure and assembly of the A-C linker connecting microtubule triplets in centrioles

**DOI:** 10.1126/sciadv.ady3689

**Published:** 2025-10-08

**Authors:** Bin Cai, Jingwei Xu, Erik H. Collet, Ellen Aarts, Leo Luo, Alexander Leitner, Takashi Ishikawa, Pedro Beltrao, Chad G. Pearson, Martin Pilhofer, Michal Wieczorek

**Affiliations:** ^1^ Institute of Molecular Biology and Biophysics, Department of Biology, ETH Zürich, Zürich, Switzerland.; ^2^ University of Colorado Anschutz School of Medicine, Aurora, CO, USA.; ^3^ Institute of Molecular Systems Biology, Department of Biology, ETH Zürich, Zürich, Switzerland.; ^4^ Center for Life Sciences, Paul Scherrer Institute, Villigen, Switzerland.; ^5^ Department of Biology, ETH Zürich, Zürich, Switzerland.

## Abstract

Centriole assembly involves the coordination of centriolar modules. One module is the A-C linker, an enigmatic protein assembly connecting the A-microtubule of one microtubule triplet to the C-microtubule of the neighboring triplet. Here, we integrated biochemistry, multiscale cryo–electron microscopy, and AlphaFold modeling to investigate the architecture of the centriole. Using an improved centriole isolation method, we determined the structure of the A-C linker bound to microtubule triplets, which revealed how the A-C linker cross-links microtubules and integrates with the B-C junction. We found marked changes in the structure and composition of the A-C linker that correlate with the presence of other centriolar modules, including the pinhead, cartwheel, and inner scaffold. Our findings show that the A-C linker is a highly integrated component of the centriole whose polymorphism may orchestrate the assembly of spatially distinct centriolar modules, and provide a framework for dissecting the biology of centrioles.

## Teaser

Multiscale cryo-EM of centrioles reveals the structure and polymorphism of the A-C linker connecting microtubule triplets.

## INTRODUCTION

Centrioles are evolutionarily conserved organelles essential for the formation of centrosomes and cilia (*1*). The structure and function of the centriole have been investigated using light microscopy as early as the 19th century (*2*, *3*). More recently, electron microscopy (EM) imaging has revealed that the centriole is a ninefold symmetrical, microtubule-based assembly (*4*). Centrioles are polarized structures, and their polarity is defined by the relative orientation of their microtubule minus and plus ends. A hallmark of the centriole’s minus end is the presence of microtubule triplet (MTT) structures (with A-, B-, and C-microtubules), which transition into microtubule doublets (with only A- and B-microtubules) toward the centriolar plus end (*5*).

The organization of microtubule components in centrioles is coordinated by an extensive yet poorly understood set of protein assemblies, which we call “centriolar modules” (*6*). Toward the minus end of the centriole, a so-called “A-C linker” bridges two adjacent MTTs by connecting the A-microtubule of one MTT with the C-microtubule of the neighboring MTT (*4*). The A-C linker displays a striated structure that begins at the centriole’s minus end and extends toward its plus end (*7*). The structure and function of the A-C linker are also coordinated with other centriolar modules (*8*). Specifically, at the minus end of the centriole, the A-C linker has been reported to associate with the “pinhead” and “cartwheel” structures (hereafter, “pinhead-cartwheel”) (*9*), a radially organized centriolar module that binds to MTTs to establish their ninefold symmetric organization (*10*). Toward the centriole’s plus end, the A-C linker overlaps with another module: the inner scaffold, a lattice of unidentified proteins that lines the core of the centriolar lumen to help maintain its structural integrity (*9*, *11*). Notably, the region marked by the inner scaffold does not overlap with the one containing the pinhead-cartwheel (*9*), providing a transitional region in which the A-C linker is present but might not interact with known centriolar modules.

Previous studies using cryo–electron tomography (cryo-ET) have reported structures of several centriolar modules, including the A-C linker, at resolutions ranging from ~8 to 40 Å (*9*, *10*, *12*–*19*); however, detailed molecular models of these assemblies are still missing, in large part due to the lack of compositional information. Cell biological studies combined with ultrastructure expansion microscopy (U-ExM) imaging (*6*) have helped to assign some centriolar components [e.g., SAS-6 in the cartwheel (*6*, *20*, *21*) and POC1 to MTT junctions (*12*)], but most proposed centriolar protein candidates currently lack direct structural evidence for their roles in the centriole. Thus, despite extensive efforts to elucidate the nature of the centriole, an understanding of its molecular structure and composition is currently limited.

An in-depth characterization of centrioles has been hindered in large part by the lack of an efficient and high-yield biochemical isolation approach (*1*). Centrioles have a low cellular abundance and are tightly embedded into surrounding cytoskeletal networks (*22*, *23*) and membrane structures (*24*, *25*), and centriolar modules are connected by a complex network of uncharacterized proteins (*8*), rendering the biochemical isolation of centrioles for high-resolution structural studies challenging. Moreover, it remains unclear whether features of isolated centrioles faithfully represent their in situ architecture. A multiscale imaging approach integrating different methods is therefore needed to dissect the structure and composition of centrioles.

## RESULTS

### Biochemical isolation of *Tetrahymena thermophila* centrioles validated by in situ cryo-ET

**Fig. 1.**
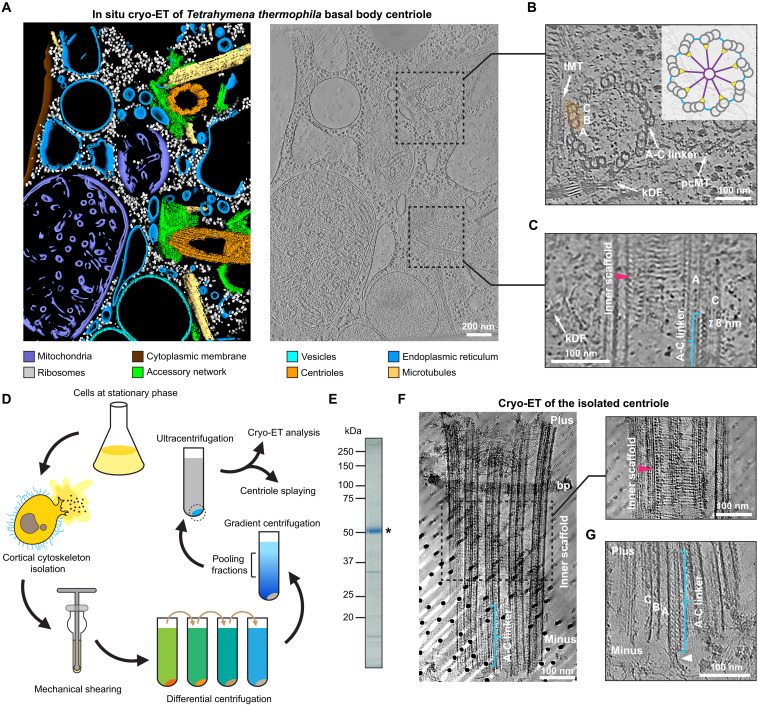
Biochemical isolation of *Tetrahymena* centrioles validated by in situ cryo-ET. (**A**) Segmented surfaces (left) and slice through the corresponding cryo-tomogram (right; 1.8-nm projection) of a FIB-milled *Tetrahymena* cell, showing two centrioles. Centrioles are highlighted with dashed boxes, and zoomed-in and reoriented views are shown in (B) and (C). (**B** and **C**) Zoomed-in cross-sectional (B) and transverse (C) views of the centrioles shown in (A). The A-C linker (8-nm repeat indicated), inner scaffold (indicated by a magenta-colored arrowhead), an MTT (highlighted in orange) and its microtubule constituents (“A”-, “B”-, and “C”-microtubules), the kinetodesmal fiber (kDF), transverse microtubules (tMT), and postciliary microtubules (pcMT) are labeled. (**D**) Schematic of *Tetrahymena* centriole isolation scheme for downstream structural analysis. (**E**) Coomassie-stained SDS–polyacrylamide gel electrophoresis (SDS-PAGE) of isolated centrioles. The major protein band corresponding to tubulin (single asterisk) is indicated. (**F**) Slice through a cryo-tomogram (64-nm projection) of an isolated centriole, showing that centriole components were generally preserved after isolation. The A-C linker and basal plate (bp) and plus-end, central, and minus-end portions of the centriole are indicated. The central part of the centriole core is indicated by a dashed box, and a corresponding transverse view at a different *z*-height (1.1-nm projection) is shown on the right, highlighting the presence of the inner scaffold (magenta-colored arrowhead). (**G**) Slice through a cryo-tomogram (1.1-nm projection) of an isolated centriole, viewing the minus-end region. The A-C linker and MTT A-, B-, and C-microtubules are indicated. The A-C linker–associated filament is indicated by an arrowhead.

To decipher the molecular organization of centrioles, we first investigated the native state of centrioles in the ciliate *Tetrahymena thermophila* (hereafter, “*Tetrahymena*”). We performed an in situ analysis using cryo-ET imaging of cryo–focused ion beam (cryo-FIB)–milled lamellae of *Tetrahymena* cells (fig. S1A). The resulting cryo-tomograms revealed ultrastructural details of intracellular components such as mitochondria, ribosomes, endoplasmic reticulum, and centriole-containing ciliary basal bodies (hereafter, “centrioles,” for simplicity) (Fig. 1, A and B; fig. S1, B and C; and movie S1). Centriolar modules were also discernible in cryo-tomograms, including the transverse and postciliary microtubules and the kinetodesmal fiber, as well as the pinhead-cartwheel, the inner scaffold, and the A-C linker (Fig. 1, B and C, and fig. S1, B and C). In transverse views, the inner scaffold appears as a periodic helical assembly lining the centriolar lumen, while the A-C linker corresponds to a striated structure located between MTTs (Fig. 1C).

In situ cryo-ET is a relatively low-throughput technique, and it remains extraordinarily challenging to achieve resolutions needed for unambiguous protein identification using this method (*26*, *27*). Therefore, using the in situ data as a reference for native centriolar features, we next sought to isolate centrioles from *Tetrahymena* cells for further structural analysis. We developed an isolation strategy based on our observation that mechanical homogenization of *Tetrahymena* cells can efficiently release centrioles from their cortical cytoskeletons; centrioles can then be further purified by differential centrifugation, followed by rate-zonal centrifugation through a linear density gradient (Fig. 1, D and E, and fig. S1D) (*28*). Negative stain EM of centrioles isolated using this method confirmed key structural features (fig. S1E) (*7*), and mass spectrometry (MS) detected hallmark centriolar proteins (tables S5 to S7) (*29*). Our strategy yields quantities of centrioles sufficient for proteomic and EM analysis from as little as 50 ml of *Tetrahymena* culture, thereby making it an attractive approach for investigating the biology of centriole components.

We next examined our isolated centrioles by cryo-ET, which revealed the presence of nine microtubule-based assemblies (fig. S1F), consistent with centrioles in in situ cryo-tomograms (Fig. 1, B and C, and fig. S1, B and C). The pinhead, inner scaffold, and A-C linker modules were also observed (Fig. 1, F and G, fig. S1F, and movie S2). Unexpectedly, small segments of A-microtubule minus ends, which are capped by structures resembling γ-tubulin ring complexes (*30*–*33*) in in situ cryo-tomograms (fig. S1C), were absent in isolated centrioles (fig. S1G). We also observed a “central filament” that forms part of the A-C linker and protrudes from the minus end and away from the centriole (Fig. 1G and fig. S1G). These observations suggest that MTT-associated protein assemblies, including the A-C linker, might connect centrioles with other cellular structures, such as the cortical cytoskeleton (*23*).

### Cryo-EM of isolated centrioles reveals the architecture of the A-C linker and its connection to MTTs

**Fig. 2.**
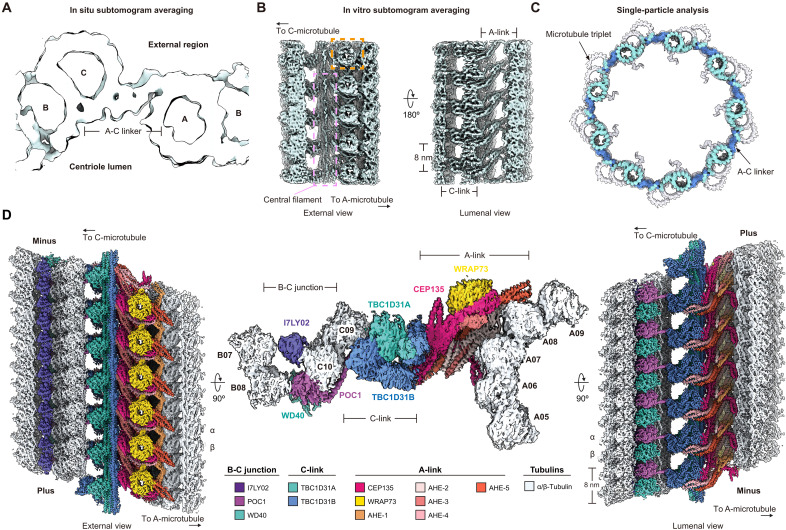
Cryo-EM of isolated centrioles reveals the architecture of the A-C linker and its connection to MTTs. (**A**) Cross-sectional view of the STA reconstruction of the centriole in cryo-FIB–milled lamellae of *Tetrahymena*. The A-C linker and MTT A-, B-, and C-microtubules are indicated. (**B**) Transverse views of the STA reconstruction of the A-C linker in isolated centrioles. The A- and C-links, as well as their 8-nm repeating nature, are indicated; a prominent β-propeller domain is highlighted by an orange dashed box; and a central filament is indicated and further highlighted by a pink dashed box. (**C**) Cross-sectional view of the single-particle analysis (SPA) reconstruction of the A-C linker (dark blue) and MTTs (light blue) in isolated centrioles, which is superimposed by a previously reported STA reconstruction (EMD-42776; shown in transparent surface representation) (*12*). Nine copies of the reconstructed maps were symmetrically assembled to represent the overall structure of the centriole. (**D**) External (left), cross-sectional (middle), and centriolar lumenal (right) views of the SPA A-C linker composite map, including adjacent MTT regions. A-C linker subregions (A- and C-links), the B-C junction, A-C linker protein components, and A-, B-, and C-microtubule protofilaments are indicated and colored according to the legend at the bottom.

Having visualized features of isolated *Tetrahymena* centrioles, we next focused on identifying centriolar modules that would be suitable for averaging-based structure determination. The presence of a striated structure in the A-C linker made it an attractive candidate. We therefore used subtomogram averaging (STA) analysis to resolve the molecular details of the A-C linker and its connection to MTTs. As a control, we first performed STA analysis on the A-C linker attached to MTTs from cryo-tomograms of cryo-FIB–milled cells (Fig. 2A and fig. S2A). This resulted in a ~30-Å resolution reconstruction with qualitatively similar features as previous STA reconstructions of centrioles (*9*, *10*, *12*–*19*).

Using the consensus in situ reconstruction of the *Tetrahymena* A-C linker as a three-dimensional (3D) reference, we next performed STA analysis on the A-C linker from isolated centrioles (fig. S2B). Unexpectedly, 3D classification revealed two main classes for the A-C linker (“class 1” and “class 2”; fig. S2, B to D). Similar structural diversity in the A-C linker was also observed after 3D classification of the in situ STA data (fig. S2A). Moreover, the particles from A-C linker class 1 could be mapped to a region covering ~100 nm of the isolated *Tetrahymena* centriole beginning from the very minus end, while particles from A-C linker class 2 mapped mainly to an adjacent region spanning between ~100 to 200 nm from the centriole’s minus end (fig. S2E), suggesting that the A-C linker adopts different morphologies depending on its location along the centriole. Because of its initial higher quality reconstruction, class 1 was first selected for further structural analysis.

STA analysis was used to generate a 7.3-Å resolution class 1 reconstruction (Fig. 2B, fig. S2C, and table S1), which revealed clear structural details in the A-C linker, including recognizable secondary structure elements (Fig. 2B and fig. S2, C and D). Overall, the STA reconstruction shows that the *Tetrahymena* A-C linker forms a repeating structure with 8-nm periodicity. The A-C linker stretches from the A-microtubule of one MTT (via an “A-link”) to the C-microtubule of the neighboring MTT (via a “C-link”) (Fig. 2B) (*10*). The A-link is composed of an α-helical network decorated by a doughnut-shaped density facing the external side of the centriole (Fig. 2B and fig. S2D). The C-link is made of globular domains, and the A- and C-links are connected to one another by the central filament (Fig. 2B), as evidenced in cryo-tomograms of isolated centrioles (Fig. 1G and fig. S1G).

While the STA reconstruction of the A-C linker in isolated centrioles revealed unprecedented structural detail, its resolution was insufficient to confidently identify proteins. To achieve higher resolutions that could facilitate protein assignment and modeling, we therefore sought to perform single-particle analysis (SPA) of isolated centrioles. Cryo-EM imaging of the entire barrel-shaped centriole was not trivial and led to overlapping centriolar module particles in cryo-EM micrographs (fig. S3A), limiting their contrast and alignment accuracy. We therefore reasoned that it would be necessary to splay the centriole into structures thin enough for SPA. We found that mechanical disruption by an additional round of ultracentrifugation was a relatively simple yet effective strategy for splaying isolated centrioles (see Materials and Methods). Mechanically disrupted centrioles were vitrified and imaged by cryo-EM, revealing that isolated centrioles became separated into MTTs retaining various combinations of A-, B-, and C-microtubules interconnected by A-C linkers (fig. S3B). Using the STA reconstruction from isolated centrioles above as a reference (Fig. 2B), SPA improved the resolution of the A-C linker class 1 reconstruction to between 3.6 and 3.8 Å (Fig. 2, B to D, figs. S3 and S4, and tables S2 and S3). Moreover, expanding our refinements into regions of MTTs adjacent to the A-C linker allowed us to resolve several additional centriolar modules, namely, the centriolar B-C inner junction (hereafter, the “B-C junction”); a 16-nm repeat average of the A-microtubule, which appears to display qualitatively similar microtubule inner protein (MIP) decorations as the A-microtubule in *Tetrahymena* axonemal doublets (*34*); and the centriolar A-B inner junction (hereafter, the “A-B junction”) (Fig. 2, C and D, and fig. S4). These SPA density maps allowed us to identify proteins and build a model for the A-C linker connected to MTTs (Fig. 2D).

### The C-link is composed of POC1 and TBC1D31-like proteins

**Fig. 3.**
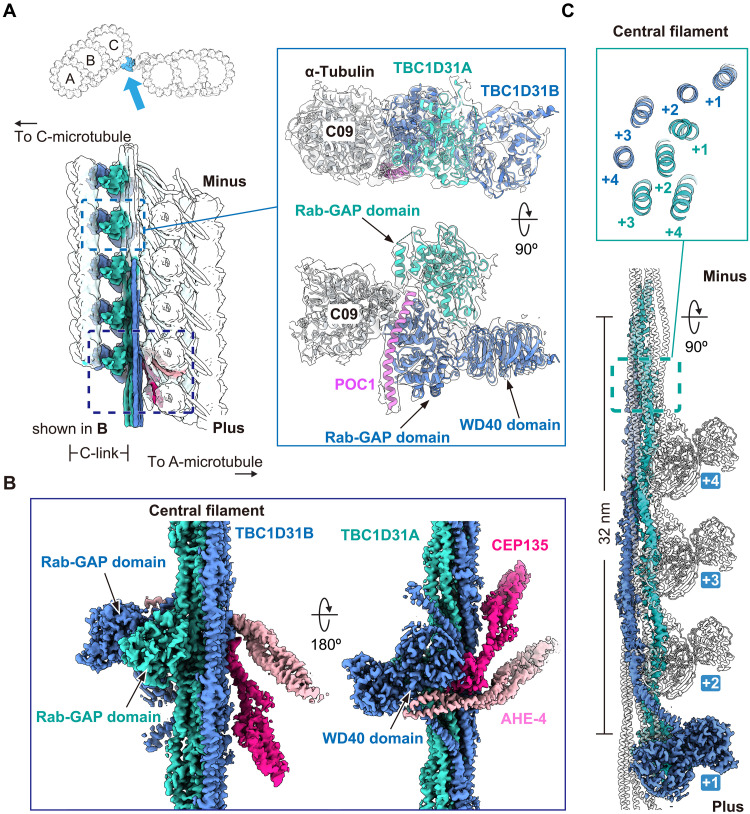
The C-link is composed of POC1 and TBC1D31-like proteins. (**A**) Zoomed-in view of cryo-EM densities for α-tubulin, POC1’s C-terminal α helix, and TBC1D31 (TBC1D31A/B) proteins in the C-link of the A-C linker. A schematic of the C-link is shown on the left for reference. (**B**) Segmented cryo-EM density showing the organization of TBC1D31A/B in the central filament together with the N terminus of CEP135 and AHE-4. (**C**) Combination cryo-EM density (colored) and model (white cartoon representation) of the central filament built from multiple copies of TBC1D31A/B. The length of four 8-nm repeats (32 nm in total) that are needed to contribute all eight α helices in the heterooctameric central filament (inset view) is indicated.

To identify protein subunits in the A-C linker SPA reconstruction, we generated AlphaFold2 models of proteins derived from MS analysis of isolated centrioles (tables S5, S6, and S7). This procedure identified two proteins in the C-link of the A-C linker, corresponding to UniProt accession IDs Q23AD1 and Q23K58. Both *Tetrahymena* proteins are homologs of human TBC1D31 (also called WDR67), which was previously proposed to be a component of both the A-C linker and of centriolar satellites (*6*, *35*–*37*). Both proteins are also predicted to contain a Rab guanosine triphosphatase activation protein (Rab-GAP) domain sandwiched between an N-terminal WD40 domain and a long C-terminal α helix. AlphaFold2 Multimer predictions suggested that the Rab-GAP domains of Q23AD1 (hereafter, “TBC1D31A”) and Q23K58 (hereafter, “TBC1D31B”) can form a complex together with the C-terminal α helix of POC1, a previously described component of *Tetrahymena* MTT inner junctions (*12*). We next used the software COLORES to perform unbiased global fitting of the predicted POC1 and TBC1D31A/B-containing subcomplex into our SPA maps. The automated fitting procedure showed that this subcomplex agrees well with the C-link density (fig. S5, A and B) (*38*, *39*), indicating that TBC1D31A/B and POC1 form part of the C-link and connect the A-C linker with α-tubulins in protofilament C09 of the C-microtubule (Fig. 3A).

Consistent with STA analysis, the SPA reconstruction showed that the C-link is connected to the A-link via the central filament. The WD40 domain of TBC1D31B and the central filament bridge the C-link with the A-link by connecting to coiled-coil domains stemming from an A-link–derived α-helical element (AHE; AHE-4), as well as from CEP135 (Fig. 3B), whose assignment is detailed further below. The improved resolutions also revealed that the central filament is a continuous bundle of long α helices formed by the C-terminal α-helical regions of TBC1D31A/B (Fig. 3, B and C). Each TBC1D31A/B C-terminal α helix spans ~32 nm in the central filament, which corresponds to the length of four α/β-tubulin dimers. A cross section of the TBC1D31A/B-containing central filament shows that eight laterally associated α helices (four from TBC1D31A and four from TBC1D31B) are organized in a staggered arrangement relative to one another. Notably, as the central filament extends past the minus end of the centriole, it would necessarily have a maximum length of ~32 nm, which matches the lengths of the central filament protrusions we observed in cryo-tomograms of isolated centrioles (Fig. 1G and fig. S1G). U-ExM of *Tetrahymena* cells expressing TBC1D31A/B fused to green fluorescent protein (GFP) at their C termini revealed similar localization of both proteins to the proximal ends of MTTs (fig. S6, A to D), further supporting their assignment to the C-link and the central filament structure.

### The C-link, B-C junction, and A-B junction are integrated together via POC1

**Fig. 4.**
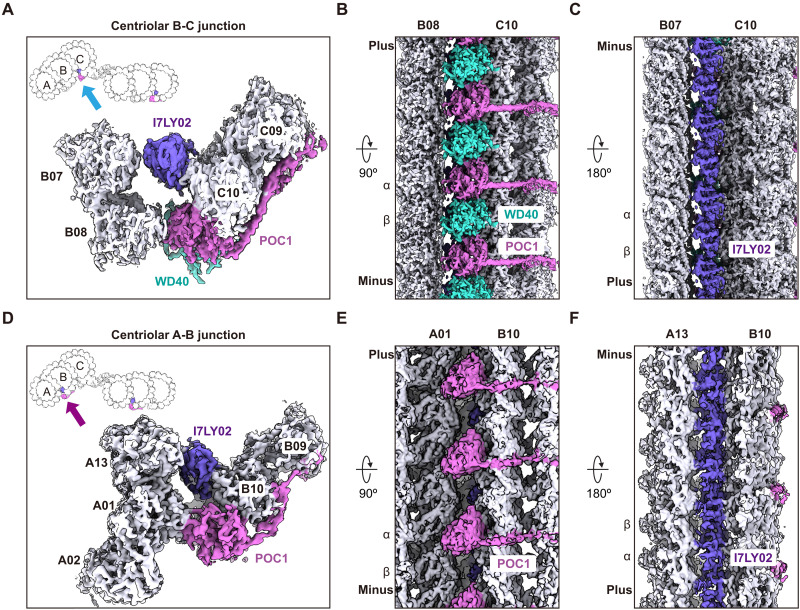
The C-link and B-C junction are integrated via POC1. (**A** to **C**) Cross-sectional (A), external (B), and centriolar lumenal (C) views of the centriolar B-C junction cryo-EM densities. POC1, the WD40 domain ladder, the I7LY02 ladder, B- and C-microtubule protofilaments, and α- and β-tubulins are indicated. A schematic is shown on the top left in (A). (**D** to **F**) Cross-sectional (D), external (E), and centriolar lumenal (F) views of the centriolar A-B junction cryo-EM densities. POC1, the I7LY02 ladder, A- and B-microtubule protofilaments, and α- and β-tubulins are indicated. A schematic is shown on the top left in (D).

By tracing the density of the POC1 α helix in the C-link, we found that it leads to a WD40 domain bridging protofilament B08 in the B-microtubule with protofilament C10 of the C-microtubule. Automated fitting of an AlphaFold2 Multimer prediction containing POC1 together with α/β-tubulin suggested that this density corresponds to POC1’s N-terminal WD40 domain (Fig. 4, A and B, and fig. S5, A and C), consistent with previous reports (*12*). We observed an additional WD40 domain sandwiched between successive POC1 proteins in the B-C junction. Although we left it unassigned in this study due to insufficient map quality in this region (Fig. 4, A and B), we hypothesize that the density could correspond to the WD domain of TBC1D31A based on its proximity to TBC1D31A, which was not found in the C-link.

In addition to POC1 and the unassigned WD40 domain, we observed an array of α-helical domains lining the lumen of the C-microtubule (dark purple densities in Fig. 4A). The domains in this array bridge protofilament C10 of the C-microtubule with protofilament B07 of the B-microtubule and exhibit a ~4-nm repeat, corresponding to one domain binding each longitudinally associated α- and β-tubulin monomer. Automated fitting of our *Tetrahymena* centriolar AlphaFold2 model library revealed that these α-helical densities correspond to I7LY02, a protein with no obvious homology outside of the phylum Ciliophora (Fig. 4, A and C, and fig. S7).

We also identified a second POC1- and I7LY02-containing array of proteins in the A-B junction of MTTs (Fig. 4, D to F, and fig. S8, A and B), consistent with similar, but unassigned, densities reported recently (*12*, *40*). Although POC1 and I7LY02 occupy the same respective binding sites in the A-B junction as the PACRG/CFAP20 and I7M279/CFAP52/IJ34 arrays found in axonemal microtubule doublets (*34*), their structures and microtubule-binding modes are entirely different (fig. S8, A to D). This suggests that MTTs may have evolved distinct strategies to stabilize junctions between their partial microtubule lattices.

### The A-link is composed of WRAP73, CEP135, and a network of α-helical elements

**Fig. 5.**
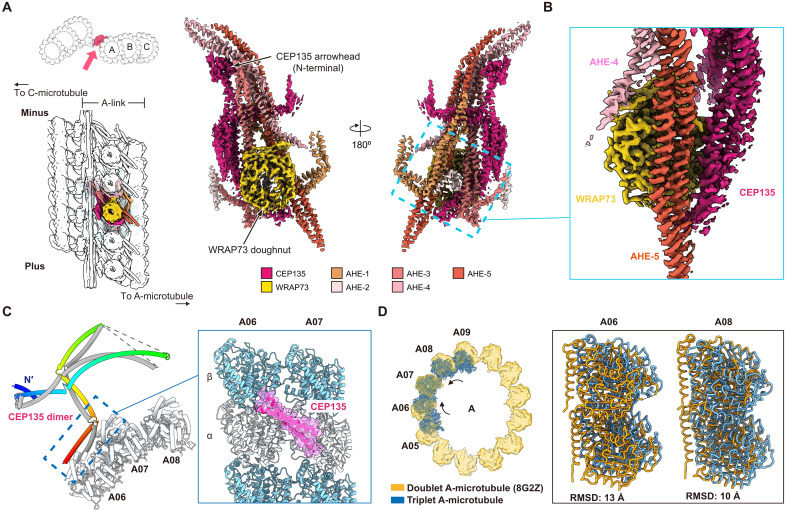
The A-link is composed of WRAP73, CEP135, and a network of α-helical elements. (**A** and **B**) Two transverse views of the A-link in the A-C linker composite cryo-EM density map, colored according to the legend at the bottom of the panel. A schematic is shown on the left for reference. The location of the interface between WRAP73, CEP135, AHE-4, and AHE-5 is outlined by a dashed box, with the corresponding zoomed-in view shown in (B). (**C**) Overview of the CEP135 dimer model (cartoon representation; one monomer in gray, another with rainbow colors from N to C terminus) and its interaction with the A-microtubule. Inset shows zoomed-in view of CEP135 residues ~325 to 360 (shown with corresponding density in transparent surface) cross-bridging A-microtubule protofilaments A06 and A07. α- and β-tubulins are colored and indicated. (**D**) Superposition of the MTT A-microtubule model (cartoon representation) with the A-microtubule in the microtubule doublet [surface representation; Protein Data Bank (PDB) ID: 8G2Z] (*34*), using protofilament A07 as an alignment anchor. Inset shows the lateral displacement of α/β-tubulin in protofilaments A06 and A08 in the MTT versus in the doublet structure, with associated root mean square deviation (RMSD) values.

Next, we focused on the A-link of the A-C linker, which consists of a complex network of α helices decorated by a nine-bladed β-propeller domain (Fig. 5A). Automated fitting of our MS-derived AlphaFold2 model library identified that the unusual nine-bladed β-propeller density can only correspond to the WD40 domain protein I7MIG5 (Fig. 5, A and B, and fig. S9). Notably, the human homolog of I7MIG5 corresponds to the centriole and centriolar satellite component WRAP73 (also known as WDR8) (*29*, *35*).

I7MIG5 (hereafter, “WRAP73”), in turn, coordinates an α-helical dimer consisting of residues ~1 to 360 of *Tetrahymena* Bld10/CEP135, which was identified from AlphaFold2 Multimer predictions as a WRAP73-interacting protein that matches the observed α-helical density (see Materials and Methods, Fig. 5, A and B, and fig. S10). This assignment was further supported by U-ExM, which localized N-terminal GFP fusions of both CEP135 gene variants (CEP135-Q22AS4 and CEP135-Q24GZ2) to the proximal ends of MTTs in *Tetrahymena* cells (see Materials and Methods and fig. S6, E to H). The N terminus of the CEP135 dimer extends away from WRAP73 and terminates at the central filament, forming contacts with the WD40 domain of TBC1D31B (Figs. 3B and 5A). WRAP73 and CEP135 associate with the A-microtubule, in part, through five unassigned AHEs (AHE-1 to AHE-5) (Fig. 5A). Structural modeling of the entire A-link revealed a hierarchical organization: AHE-1 is attached to A-microtubule protofilaments A07 and A08, followed by the sequential layering of AHE-2 to AHE-5 (fig. S11, A to E); the A-link is completed by the binding of CEP135 and WRAP73 to the top of the unassigned α-helical network (fig. S11, F and G). Notably, CEP135 contributes a short α-helical segment that forms extensive contacts with nearly all AHEs (fig. S11H), suggesting that CEP135 is a key organizer of the A-link.

In addition to the AHEs, the A-link is also connected to the A-microtubule via residues ~325 to 360 of the CEP135 dimer, which form an α-helical coiled coil that diagonally bridges protofilament A06 to protofilament A07 (Fig. 5C). Notably, this portion of CEP135 contrasts with previously reported microtubule-binding domains in both human and *Drosophila* CEP135 homologs (*41*, *42*). This discrepancy may be due to the presence of multiple microtubule-binding domains in CEP135 or the lack of other centriolar components in previously reported in vitro microtubule-binding assays (*41*, *42*). The interface between CEP135-bound A-microtubule protofilaments is altered relative to cytoplasmic microtubule singlets (*43*, *44*), as well as the A-microtubule in the *Tetrahymena* axonemal doublet (Fig. 5D) (*34*). Aligning A-microtubules from axonemal doublets and MTTs via protofilament A07 shows a substantial compaction of protofilaments A06 [root mean square deviation (RMSD): 13 Å] and A08 (RMSD: 10 Å) toward protofilament A07 (Fig. 5D). This suggests that local changes in tubulin lattice parameters may accommodate, or even be induced by, the attachment of A-C linker components to MTTs.

### The A-C linker is a polymorphic structure that is coordinated with distinct centriolar regions

Having determined the structural details of the A-C linker corresponding to class 1 in STA analysis of isolated centrioles, we next sought to decipher the architecture of the second A-C linker class [class 2 in fig. S2 (B to E)]. We first confirmed through unsupervised 3D classification that a second class of A-C linker is also present in our SPA data (fig. S3, C and D) and that class 1 and class 2 A-C linkers are structurally consistent between STA analysis and SPA (Fig. 2, B to D, and figs. S2B and S3C). Next, we mapped the respective particles from SPA class 1 and class 2 back to cryo-EM micrographs, revealing that class 1 particles localize mainly to the minus-end regions of MTTs, whereas class 2 particles are mainly found in MTT plus-end regions (fig. S3E), consistent with our STA analysis (fig. S2E).

**Fig. 6.**
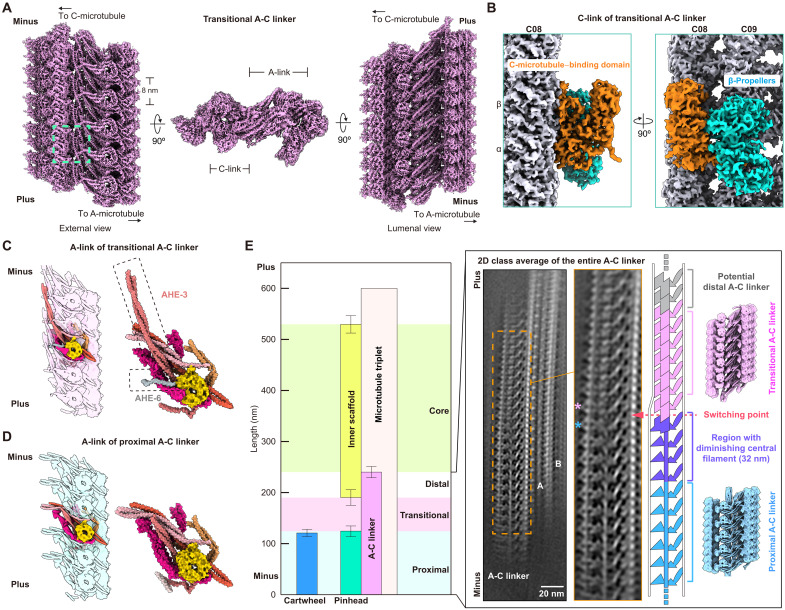
The A-C linker is a polymorphic structure coordinated with distinct centriolar regions. (**A**) External (left), cross-sectional (middle), and lumenal (right) views of the transitional A-C linker SPA composite map. The A-link, C-link, an 8-nm repeat, and plus and minus ends are labeled. A green dashed box (left) highlights the C-link, with zoomed-in views shown in (B). (**B**) Two views of the C-link in the transitional A-C linker composite map (surface representation). Globular domains comprising the C-link (C-microtubule–binding domain: orange; β-propellers: cyan) and the C-microtubule lattice (α/β-tubulin: white) are shown. (**C** and **D**) Views of A-link models from the proximal (C) and transitional (D) A-C linkers. Schematics are shown on the left for reference. Protein subunits are color coded as in Fig. 5A. The additional AHE (AHE-6: gray) and the AHE with structural differences (AHE-3: coral) are highlighted by dashed boxes. (**E**) Left: A plot of regions containing different centriolar modules in *Tetrahymena* oral apparatus cryo-tomograms (plotted values show the mean ± SD). Right: A 2D class average of the entire MTT, where the A-C linker, A- and B-microtubules, and centriolar plus and minus ends are indicated. The zoomed-in view of the region highlighted by an orange box shows the region, in which the proximal A-C linker switches into the transitional A-C linker in the 2D class average (magenta dashed arrow), and which is schematized in the cartoon drawing. The pink asterisk corresponds to the emergence of the first transitional A-C linker C-link globular domains, while the blue asterisk highlights the last TBC1D31A/B-containing C-link from the proximal A-C linker. The locations of a hypothetical distal A-C linker structure, as well as the gradually diminishing TBC1D31A/B central filament, are indicated in the schematic. Corresponding maps of the two A-C linkers identified in this study are shown on the right for reference.

We next used SPA to determine the structure of A-C linker class 2 at a resolution range of 3.8 to 3.9 Å (Fig. 6A, fig. S12, and table S4). The improved reconstructions revealed that A-C linker class 2 has a bipartite architecture comprising an A-link and a C-link (Fig. 6A), analogous to A-C linker class 1 (Fig. 2D). Both classes share a similar structural organization in the A-link (Fig. 6, A, C, and D), with minor exceptions in A-C linker class 2, including (i) an additional unassigned AHE (AHE-6) that contacts both WRAP73 and CEP135 (Fig. 6C); (ii) an altered orientation of CEP135’s N terminus (fig. S13, A and B); and (iii) CEP135’s microtubule-binding region, which is less well resolved (fig. S13B). In contrast, the C-link of A-C linker class 2 does not appear to contain either POC1 or TBC1D31A/B densities and is instead characterized by two β-propellers and an unassigned C-microtubule–binding domain (Fig. 6, A and B). We could not assign these C-link proteins but note that they repeat every 8 nm and take the place of TBC1D31A/B and the C-terminal α helix of POC1 in A-C linker class 1 (Fig. 3, A and B).

Because SPA and STA analyses indicated that A-C linker class 1 and class 2 map to spatially distinct regions of the *Tetrahymena* centriole, we next explored whether different types of A-C linkers are correlated with other centriolar modules. We therefore measured the start and end points of spatial regions occupied by different centriolar modules in cryo-tomograms of centrioles embedded in the *Tetrahymena* oral apparatus (Fig. 6E, left (*n* = 6), and table S8), which contains all centriolar modules and allows direct imaging by cryo-ET without the need for cryo-FIB milling. On the basis of the locations of class 1 and class 2 A-C linker particles in cryo-tomograms (fig. S2E), we found that A-C linker class 1 is located in a “proximal” region of the A-C linker characterized by the presence of the pinhead-cartwheel. In contrast, A-C linker class 2 is found in an adjacent region of the centriole defined by the “transition” from the pinhead-cartwheel to the inner scaffold (Fig. 6E, left). On this basis, we term the class 1 A-C linker as the “proximal A-C linker,” and the class 2 A-C linker as the “transitional A-C linker.” Further SPA analysis allowed us to observe a ~30-nm-long region, in which the proximal A-C linker becomes the transitional A-C linker (Fig. 6E, right), consistent with a gradual loss of TBC1D31A/B C-terminal α helices in the central filament. Therefore, our results demonstrate the existence of at least two compositionally and spatially distinct types of A-C linkers in *Tetrahymena* centrioles and suggest a structural basis for the transition between the two identified A-C linker types.

## DISCUSSION

We have demonstrated that the A-C linker exhibits a polymorphic architecture, whose structure and composition change in distinct regions of the centriole. Previous studies of centrioles in the evolutionarily distant species *Trichonympha* spp. (*10*, *15*), *Chlamydomonas reinhardtii* (*14*, *18*, *19*), *Paramecium tetraurelia* (*9*), *Tetrahymena* (*12*), and *Homo sapiens* (*13*) showed that they contain several distinct centriolar regions based on the relative locations of centriolar modules, namely, (i) the A-C linker, (ii) the pinhead-cartwheel, and (iii) the inner scaffold (*8*, *9*). In addition, previous STA analysis of *Paramecium* (*9*) and *Chlamydomonas* (*14*, *18*, *19*) centrioles suggested that the architecture of the A-C linker may change across these different centriolar regions. However, whether such differences could be due to variable helical twists in MTTs along the centriolar axis, or whether the A-C linker changes its conformation and/or composition (*8*), could not previously be addressed, due to resolution limitations.

Our unsupervised classification in the SPA and STA analysis of *Tetrahymena* centrioles revealed the presence of two distinct A-C linker types: the proximal A-C linker, which occupies the proximal ~100 nm of the MTTs, and the transitional A-C linker, which abruptly switches in conformation and composition and occupies a region spanning from ~100 to 200 nm from the centriole’s minus end. As confirmed by measuring the locations of different centriolar modules by cryo-ET (Fig. 6E), the *Tetrahymena* centriole can be divided into four regions relevant to our study of the A-C linker: (i) the “proximal A-C linker region,” defined by the presence of an A-C linker and the pinhead-cartwheel; (ii) the “transitional A-C linker region,” defined by the presence of an A-C linker but an absence of both the pinhead-cartwheel and the inner scaffold; (iii) the “distal A-C linker region,” defined by the presence of an A-C linker and the inner scaffold; and (iv) the “core region,” containing the inner scaffold but lacking an A-C linker (Fig. 6E). Under this scheme, structural changes in the transitional A-C linker are probably adapted to the presence or absence of neighboring centriolar modules (e.g., a missing pinhead-cartwheel in the transitional A-C linker region). Similarly, and although it was not identified in our EM analyses, we expect that an additional conformationally and/or compositionally distinct “distal A-C linker” could exist in the distal A-C linker region (Fig. 6E) and may exhibit further structural diversity to facilitate potential attachments to the inner scaffold.

Notably, our cryo-EM micrographs also showed that the A-C linker can partially dissociate from MTTs in mechanically disrupted centrioles (fig. S14, A and B). 3D classification of our SPA data revealed the presence of classes, in which only a few well-resolved MTT protofilaments remain attached to the A-C linker (fig. S14, C and D). These data show that the structure of the A-C linker remains intact even in the absence of the centriole. They also suggest that the A-C linker confers a high degree of stability to the local α/β-tubulin lattice in MTTs, inferred from its apparent ability to “strip” protofilaments away from both A- and C-microtubules.

On the basis of its exceptional stability and structural polymorphism, we propose that the A-C linker coordinates and integrates centriolar modules from beyond the proximal A-C linker region, and possibly up to the core region (Fig. 6E). Supporting this model, we found a TBC1D31A/B-derived filament bridging the A- and C-links in the proximal A-C linker that extends into the cell body (fig. S1G), which potentially interacts with the cortical cytoskeleton network (*23*). Moreover, we found that the proximal A-C linker is directly connected to the B-C junction via POC1 (Figs. 3A and 4A). Last, we found that the N-terminal portion of CEP135 forms a major part of the A-link in both types of A-C linkers. In addition to our U-ExM localization data (fig. S6, E to H), this assignment is supported by previous reports that an antibody recognizing the N terminus of CEP135 localizes the protein in between MTTs at the minus ends of centrioles (*6*) and that human WRAP73 and CEP135 form a potential complex in cells (*45*, *46*). Notably, the C-terminal portion of CEP135 was previously proposed to form part of the pinhead-cartwheel modules and a “triplet base” that connects the pinhead to the A-C linker (*47*, *48*), both of which are located more toward the centriole lumen. This suggests that different regions of CEP135—an up to ~1400–amino acid–long coiled-coil protein—function together to connect distant centriolar modules, as previously proposed (*6*, *8*, *47*–*50*). Our work not only shows how the A-C linker forms a key linkage between adjacent MTTs but also provides a structural basis for its likely interaction with other centriolar modules (e.g., pinhead-cartwheel and triplet base), allowing it to form a highly integrated centriolar module “hub.”

Despite the A-C linker’s structural polymorphism, we note that both A-C linker types identified here still exhibit general architectural principles conserved across evolutionarily divergent species (*9*, *10*, *12*–*15*, *17*), with the main goal of ensuring the cohesion of two adjacent MTTs via similar binding profiles (fig. S15, A to F). In all previously reported reconstructions containing the A-C linker (*9*, *10*, *12*–*16*), an A-link attaches to A-microtubule protofilaments ~A09 to A07, whereas a C-link interacts with C-microtubule protofilaments ~C08 to C09 from the adjacent MTT. These architectural similarities suggest compositional homology at the protein level. Except for the B-C junction protein I7LY02, the A-C linker proteins modeled in this study (WRAP73, CEP135, TBC1D31, and POC1) are highly conserved across model organisms. Moreover, mutations in the human homologs of WRAP73, CEP135, and POC1 are found in patients displaying microspherophakia (*51*), microcephaly (*52*), and primordial dwarfism (*53*), and the overexpression of CEP135 causes chromosome segregation defects in breast cancer cells (*54*). Together, our findings demonstrate that the A-C linker is architecturally and biochemically conserved, reinforcing its critical role in the centriole and providing a potential path to understanding how defects in centriole integrity might be linked to human diseases, such as cancer and ciliopathies (*51*, *53*, *55*–*57*).

Although the biological significance of centrioles was first noted over a century ago (*2*, *3*, *58*), their molecular characterization has been hindered by their immense structural complexity and the lack of reliable biochemical isolation methods. Using the model ciliate *Tetrahymena*, we have developed a high-yield centriole isolation method, validated through comprehensive in situ cryo-ET and STA analysis, and further characterized the structure of the A-C linker by leveraging cryo-EM SPA of mechanically disrupted centrioles. Moreover, adapting and applying an integrated “visual proteomics” method (*38*) allowed us to successfully identify and build models for several centriolar proteins. Our approach serves as a benchmark for the structural characterization of centrioles and will be useful for investigating other centriolar modules in *Tetrahymena* and in centrioles from diverse species in the future.

## MATERIALS AND METHODS

### Isolation of centrioles from *Tetrahymena*

*Tetrahymena* strain SB715 (TSC_SD01508, Tetrahymena Stock Center) was grown in modified Neff medium [0.25% (w/v) protease peptone, 0.25% (w/v) yeast extract, 0.5% (w/v) glucose, and 33.3 μM FeCl_3_] at room temperature. To isolate centrioles, 50 ml of culture at the stationary phase was first chilled on ice to allow the sedimentation of *Tetrahymena* cells. Afterward, cells were harvested by centrifugation at ×300*g* and 4°C for 5 min in a conical tube. All the following steps were performed on ice. Pelleted cells were resuspended gently in 5 ml of 0.25 M sucrose solution, and the cell suspension was transferred to a glass beaker in an ice-water bath and stirred gently with a magnetic stirrer. A total of 15 ml of hyperosmotic solution [10 mM tris-HCl (pH 9.0), 1 M sucrose, 1 mM EDTA, and 1 mM dithiothreitol (DTT)] was added to the cell suspension with stirring. After 30 s, 2.5 ml of detergent solution (10% (v/v) Triton X-100) was added, and the suspension was stirred for an additional 30 s. The suspension was then transferred to a 50-ml conical tube and centrifuged at ×4000*g* and 4°C for 20 min. After discarding the supernatant, the flocculent precipitate containing cortical cytoskeleton at the bottom of the tube was resuspended in 10 ml of washing solution [100 mM sodium phosphate buffer (pH 6.9) and 2 mM DTT] with a Pasteur pipette. The suspension was transferred to 1.5-ml microcentrifuge tubes and centrifuged at ×17,000*g* and 4°C for 10 min. After gentle washing with 10 mM Hepes (pH 7.5) without disturbing the pellet, the pellet was resuspended in 5 ml of 10 mM Hepes (pH 7.5) buffer. The suspension was transferred to a glass dounce homogenizer and vigorously homogenized with the B-pestle for 5 min. The homogenate was transferred to 1.5-ml microcentrifuge tubes and fractionated by differential centrifugation (×700, ×1000, ×2000, and ×17,000*g* for 5 min at 4°C accordingly). After centrifugation at ×17,000*g*, the pellet was resuspended in 1 ml of 10 mM Hepes (pH 7.5), and additional homogenization was performed with 30 strokes with the B-pestle of a glass dounce homogenizer. The homogenate was fractionated by rate-zonal centrifugation through a linear glycerol gradient [20 to 50% (v/v)]. In detail, after setting up the gradient using a Gradient Master 107 (BioComp Instrument), 600 μl of solution was removed from the top of the gradient, and 1 ml of the homogenate was loaded. Rate-zonal centrifugation was performed at ×12,000 rpm for 35 min at 4°C using an SW 55 Ti rotor (Beckman Coulter). After centrifugation, the gradient was fractionated and checked by SDS–polyacrylamide gel electrophoresis (SDS-PAGE) and negative staining EM. Fractions containing isolated centrioles were pooled and diluted with 10 mM Hepes (pH 7.5) buffer. The diluted solution was concentrated by ultracentrifugation using an SW 32 Ti rotor (Beckman Coulter) at ×100,000*g* and 4°C for 1 hour. The supernatant was carefully discarded, and the pellet was resuspended in 10 mM Hepes (pH 7.5) buffer. The suspension was imaged by negative staining EM and used for downstream cryo-sample preparation.

The splaying of the barrel-shaped centrioles was performed by an additional round of ultracentrifugation using a TLA-120.2 rotor (Beckman Coulter) at ×100,000 rpm and 4°C for 1 hour. The pellet was resuspended in 10 mM Hepes (pH 7.5) buffer and used for plunge freezing and SPA.

### Isolation of the *Tetrahymena* oral apparatus

*Tetrahymena* strain SB715 (TSC_SD01508, Tetrahymena Stock Center) was cultured under the same conditions as above. Oral apparatuses were isolated from 50 ml of culture at the stationary phase according to a previously reported method (*59*). *Tetrahymena* cells were collected by centrifugation at ×4000*g* and 4°C for 5 min in a 50-ml conical tube, and all the following steps were performed on ice. A total of 5 ml of 0.1% (v/v) Triton X-100 solution was added to cell pellets, and the mixture was stirred rapidly with a spatula for 2 min to lyse the cells. The lysate was diluted using 40 ml of ice-cold deionized water and transferred to a glass dounce homogenizer, where the lysate was homogenized vigorously with B-pestle for 5 min. The homogenate was then transferred to a 50-ml conical tube and underlaid with 2 ml of cushion solution [1 M sucrose and 0.01% (v/v) Triton X-100] using a long sampling needle. After centrifugation at ×2000*g* and 4°C for 15 min, the supernatant was discarded, and only ~1 ml of residue cushion solution was left in the tube. A total of 20 ml of 0.01% (v/v) Triton X-100 solution was added, and the pellet was resuspended with a Pasteur pipette. The resuspension was subjected to a second round of centrifugation (×2000*g* and 4°C for 15 min) as above with 2 ml of cushion solution. The supernatant was discarded after centrifugation. The pellet was resuspended in 1 ml of 10 mM Hepes (pH 7.5) buffer, transferred to a 1.5-ml microcentrifuge tube, and centrifuged at ×700*g* and 4°C for 5 min. The pellet was collected and resuspended in 200 μl of 10 mM Hepes (pH 7.5) buffer for EM analysis.

### Plunge freezing

Vitrification of *Tetrahymena* cells was done using a Vitrobot Mark IV (Thermo Fisher Scientific). To ensure that the cells were fully vitrified, the culture was grown to the stationary phase in modified Neff medium and transferred to 10 mM Hepes (pH 7.5) buffer for 2 days before collection to reduce the cell size by starvation. The starved cells were harvested and stained using carboxyfluorescein diacetate succinimidyl ester (CellTrace CFSE, Thermo Fisher Scientific). Plunge freezing was performed at 8°C with 100% humidity. A total of 4 μl of cell suspension was applied to negatively glow-discharged EM grids (R2/2 Cu 200 mesh, “specially treated,” Quantifoil). Grids were manually blotted from the back side and plunged into a liquid ethane-propane mixture [37% (v/v) ethane (*60*)]. Cryo-samples were screened using a cryo-light microscope (Zeiss LSM 900 equipped with Airyscan 2 detector and a Linkam CMS196V3 cryo-stage), and grids with good ice were used for downstream cryo-FIB milling.

For vitrification of nondisrupted, isolated centrioles or oral apparatuses for STA, 5 μl of sample was supplemented with 1 μl of 10-nm bovine serum albumin (BSA)–coated colloidal gold particles (Cytodiagnostics), and 4 μl of sample was applied onto negatively glow-discharged EM grids (R2/2 Cu 200 mesh, specially treated, Quantifoil) coated with a 2-nm-thick continuous carbon layer in a Vitrobot (8°C and 95% humidity). After a 60-s incubation time, grids were blotted for 4 to 8 s and then plunge frozen into 37% ethane-propane mixture.

For SPA of mechanically disrupted, isolated centrioles, a multiapplication method was used to increase the particle number. Briefly, the tweezers holding a negatively glow-discharged EM grid (R3.5/1 Cu 200 mesh, 2-nm continuous carbon, Quantifoil) were chilled on a metal block placed on ice outside of the Vitrobot. A total of 4 μl of splayed centriole sample was applied onto the grid and incubated for 1 min under ambient humidity. The sample was manually blotted away with filter paper (Grade 595, Ted Pella), and another 4 μl of sample was applied immediately to avoid drying of the grid. The sample was applied for a total of four times, and after blotting away the fourth application, 3 μl of sample was applied. The tweezers holding the EM grid were then mounted in the Vitrobot and blotted for 8 s at 8°C and 100% humidity and then plunge frozen into a 37% ethane-propane mixture. All grids were stored in liquid nitrogen until imaging.

### Cryo-FIB milling

Cryo-FIB milling of *Tetrahymena* cells was performed using a Crossbeam 550 FIB-SEM instrument (Zeiss) equipped with an SE2 detector, an in-lens secondary electron detector, a copper band–cooled mechanical cryo-stage (Zeiss), and an integrated vacuum transfer system (VCT500 from Leica). The general cryo-FIB milling workflow was performed as previously reported (*61*). Briefly, grids were first assembled into FIB milling Autogrids (Thermo Fisher Scientific) and mounted onto a cryo-FIB AutoGrid holder (Leica) (*62*) in a Vacuum Cryo Manipulation loading station (Leica). The AutoGrid holder was transferred into an ACE600 (Leica) for cryo–sputter coating with a 4-nm tungsten layer and was then loaded into the Crossbeam 550 using the VCT500 shuttle. Afterward, grids were coated with an extra layer of organoplatinum. Cells were targeted in scanning electron microscopy/FIB views (fig. S1A), and automated sequential FIB milling was then set up. The milling pattern aimed to generate ~250-nm-thick lamellae using four currents (rough milling: 700, 300, and 100 pA; polishing: 50 pA). After milling, the AutoGrid holder was unloaded and was transferred back to the VCM to unload grids, which were stored in liquid nitrogen before cryo-ET imaging.

### Cryo-ET data collection, cryo-tomogram reconstruction, and segmentation

All cryo-ET datasets were collected using SerialEM (*63*) on a Titan Krios transmission electron microscope G4 (Thermo Fisher Scientific) operating at 300 kV and equipped with a BioContinuum imaging filter and a K3 direct electron detector (Gatan).

For the lamella dataset, tilt series were collected using PACE-tomo (*63*) in a dose-symmetric scheme with an angular range of +44° to −66° relative to the lamellae, at a defocus ranging from −3 to −5 μm. The datasets were collected at a nominal magnification of ×19,500 (an effective pixel size of 4.51 Å per pixel) with 3° increments. The accumulated dose was ~130 e^−^/Å^2^ per tilt series.

For isolated centrioles, the targets were identified at low magnification, and the images at low and high magnification were set up as a reference for target alignment before tilt-series collection using SerialEM GUI. Tilt series were collected in a dose-symmetric scheme covering an angular range of +60° to −60° with a total electron dose of ~130 e^−^/Å^2^. The datasets were collected at a nominal magnification of ×33,000 (an effective pixel size of 2.68 Å per pixel) using 3° increments, at a defocus ranging from −3 to −5 μm.

For isolated oral apparatus, tilt series were collected using PACE-tomo in a dose-symmetric scheme with an angular range of +60° to −60°, a total dose of ~130 e^−^/Å^2^, and at a defocus ranging from −3 to −5 μm. The datasets were collected at a nominal magnification of ×63,000 (an effective pixel size of 1.38 Å per pixel) with 3° increments.

Tilt series were motion corrected using alignframes, and cryo-tomograms were manually reconstructed at a binning factor of 4 (in situ dataset and isolated centrioles) or 6 (isolated oral apparatus) using the IMOD package (*64*). Cryo-tomogram contrast was further improved by DeepDeWedge (*65*) (cryo-tomograms of FIB-milled lamellae of *Tetrahymena* cells) or cryoCARE (*66*, *67*) (cryo-tomograms of isolated centrioles and isolated oral apparatus). The denoised cryo-tomogram of a FIB-milled *Tetrahymena* cell in Fig. 1A was segmented in Dragonfly (Comet Technologies Canada; v. 2022.2) as previously described (*68*), and segmentation results were illustrated using UCSF ChimeraX (*69*).

### Subtomogram averaging

Individual MTTs were manually picked (with their minus ends as start points and their plus ends as end points) from denoised in situ cryo-tomograms using 3dmod. The coordinates were used for segmentation of overlapping particles with an interbox distance of 8 nm. The coordinates of segmented particles were imported into Dynamo (*70*) and used to crop particles from the denoised cryo-tomograms at a binning factor of 4 using dtcrop. The cropped particles (1809 particles from 14 cryo-tomograms) were subjected to a coarse alignment with rough angular search steps and without imposing symmetry using dcp, where a previously deposited map (EMD-4927) (*10*) was low-pass filtered to 60 Å and used as an alignment reference. The refined poses were used for particle cropping from the contrast transfer function (CTF)-corrected cryo-tomograms (phase flipped) at a binning factor of 2. The particles were then split into half-datasets using dteo, and each half-dataset was refined individually with precise angular search steps against the same reference generated from the last iteration. The resolution of the in situ MTT reconstruction was estimated from the averages of half-datasets based on the Fourier shell correlation (*71*) using relion_postprocess (*72*).

We next performed focused 3D classification of the in situ STA of the A-C linker using a Warp-RELION-M pipeline (*73*). Briefly, the raw dataset was preprocessed (including motion-correction, CTF estimation, and handedness check) in Warp, and particle coordinates determined from Dynamo analysis were converted to RELION-3.0 STAR format for downstream particle extraction at a pixel size of 10 Å per pixel. Subtomograms were then used for 3D classification without angular sampling in RELION-3.0 (*K* = 4), with a mask covering the A-C linker. Two 3D classes showed different features of the A-C linker, suggesting the structural diversity in the A-C linker persists in situ (fig. S2A).

For STA of the A-C linker in isolated centrioles, raw tilt series were preprocessed in Warp as described above (fig. S2B). The filaments were then manually picked from denoised cryo-tomograms using the “filamentWithTorsion” model in Dynamo and were segmented with an interbox distance of 8 nm. The azimuth orientations of particles were randomly assigned using dynamo_table_randomize_azimuth, and the resulting particle coordinates and poses were imported into Warp to export subtomograms at a pixel size of 10.71 Å per pixel (at a binning factor of 4). The extracted particles (42,510 particles from 175 cryo-tomograms) were subjected to one round of 3D classification without classification (*K* = 1) in RELION-3.0, with limited angular search ranges in two Euler angles (--sigma_tilt 5 and --sigma_psi 3.333). Afterward, one round of 3D classification without sampling was performed, where a local mask covering the A-C linker was used. The particles in reasonable 3D classes were combined and used to export subtomograms at a pixel size of 5.44 Å per pixel (at a binning factor of 2) in Warp. The exported particles were subjected to one round of 3D local refinement in RELION-3.0 and imported into M for initial refinement (including image warp grid, particle poses, stage angles, and volume warp grid). The refined particles were exported at a pixel size of 4.0 Å per pixel, and the poses were further refined locally in RELION-3.0. One round of focused 3D classification on the A-C linker was performed, showing two 3D classes with clear structural features. The particles from these two 3D classes were selected to perform 3D local refinement individually, revealing two different A-C linker–like structures (class 1 and class 2). The particles from these classes were each subjected to a second round of refinement (similar refinement parameters as the initial round) in M, followed by individual 3D local refinement against the corresponding 3D class models in RELION-3.0 at a pixel size of 2.68 Å per pixel (at a binning factor of 1). The refined particles were then used for another round of M-RELION refinement. A final resolution of 7.3 Å for the A-C linker in class 1 was obtained from 4656 subtomograms without imposing symmetry, whereas the A-C linker class 2 was obtained at 8.9 Å using 4175 subtomograms (fig. S2, B and C, and table S1).

### Cryo-EM SPA data collection

All cryo-EM SPA datasets were collected using EPU (Thermo Fisher Scientific) on a Titan Krios transmission electron microscope G3i (Thermo Fisher Scientific) operating at 300 kV and equipped with a BioQuantum energy filter and a K3 direct electron detector (Gatan). Automated collection was performed at a nominal magnification of ×64,000 with a pixel size of 1.34 Å per pixel. Given that this pixel size was not initially calibrated, for initial data processing, 1.34 Å per pixel was used, while a calibrated pixel size of 1.315 Å per pixel was used for postprocessing of final cryo-EM density maps. The total electron dose was 32 to 36 e^−^/Å^2^. The energy filter slit width was set to 20 eV. The defocus range was −0.8 to −3.0 μm. Data were collected in Counted Super Resolution mode, with a binning of 2 and fractions (number) of 40. The whole dataset was collected across seven individual sessions, resulting in 247,455 movies (dataset 1: 13,209 movies; dataset 2: 15,302 movies; dataset 3: 60,630 movies; dataset 4: 30,539 movies; dataset 5: 51,660 movies; dataset 6: 28,518 movies; dataset 7: 47,597 movies). In downstream processing, the data were divided into two batches, where batch 1 includes datasets 1 to 5 and batch 2 contains datasets 6 and 7. All SPA data collection and processing statistics are listed in tables S2 and S4.

### Cryo-EM SPA

Motion correction of raw movies was performed using MotionCor2 (*74*) with dose weighting. CTF estimation was performed using CTFFIIND4 (*75*). Initial data processing was performed on dataset batch 1 (171,340 movies). Motion-corrected micrographs were binned by a factor of 4 and screened manually for centriole-containing micrographs with a custom Python script. The resulting 45,755 micrographs were used for manual picking in RELION-4.0 (*76*), and filament segments were extracted with a box size of 360 pixels, an 80-Å interbox spacing, and a pixel size of 1.34 Å per pixel. A total of 2,491,506 particles was extracted using RELION-4.0 and exported to CryoSPARC v4.0 (*77*) using pyem (*78*), and 2D classification was performed to remove bad particles. The remaining particles were roughly aligned using helical reconstruction in CryoSPARC v4.0, using the STA reconstruction (class 1) of isolated centrioles in this study as a reference. Particles with refined poses were exported to RELION-5.0 (*72*) to perform 3D classification with Blush regularization (*79*), resulting in two major 3D classes: one adopting an extended conformation that was later characterized to be the proximal A-C linker and another one adopting a narrow conformation that was later characterized to be the transitional A-C linker. The narrow conformation was used as a new 3D reference for downstream processing of the entire SPA dataset.

In the image processing of the entire dataset, motion-corrected micrographs in dataset batches 1 and 2 were combined, manually filtered, and subjected to filament picking as described above. A total of 3,928,235 particles was extracted with a pixel size of 2.68 Å per pixel (at a binning factor of 2) and a box size of 180 pixels and were then exported to CryoSPARC v4.0. All particles were subjected to helical reconstruction and then exported back to RELION-5.0 for 3D classification. All 3D classifications in RELION-5.0 afterward were performed with Blush regularization. A total of 1,778,344 particles from reasonable 3D classes after the first round of 3D classification (*T* = 4) was pooled and subjected to additional two rounds of 3D classification (*T* = 6). Particles belonging to either the proximal or transitional A-C linkers were selected and reextracted in RELION-5.0 with a pixel size of 1.34 Å per pixel and a box size of 360 pixels, respectively (proximal A-C linker: 210,425 particles; transitional A-C linker: 358,439 particles).

For the transitional A-C linker, the reextracted particles were subjected to 3D classification (*T* = 6) in RELION-5.0. Particles from good 3D classes were then refined with CTF refinement and Bayesian polishing. The consensus transitional A-C linker structure was determined at a resolution of 4.0 Å from 100,116 particles without applying symmetry. We next established a focused refinement pipeline for different subregions. The consensus map was segmented into 2 subregions (A-link with A-microtubule and C-link with C-microtubule) using Segger in UCSF Chimera (*80*) for local processing, where individual masks were created. Particles were symmetrically expanded into five helical-related repeats, and density subtraction was performed using the local masks. Afterward, particles were recentered and subjected to 3D classification. For the A-link with A-microtubule, a total of 393,606 particles was used to determine its structure at a resolution of 3.9 Å (fig. S12). For the C-link with C-microtubule, 290,362 particles were used to determine its structure at a resolution of 3.8 Å. Another round of focused 3D classification on the C-link without C-microtubule was performed to resolve the C-link region, which resulted in a 3.8-Å resolution reconstruction from 104,894 particles.

For the proximal A-C linker, a similar image processing strategy was performed as for the transitional A-C linker, which resulted in a 4.1-Å resolution reconstruction of the consensus A-C linker from 155,485 particles. The consensus A-C linker was then segmented into three subregions (the A-link, the C-link, and the A-link with A-microtubule) using Segger in UCSF Chimera, and individual masks were created and applied to a similar focused refinement pipeline as described above (fig. S3). Last, different subregions were determined at resolutions ranging from 3.6 to 3.8 Å (the A-link: 3.8 Å with 279,061 particles; the A-link with A-microtubule: 3.7 Å with 695,170 particles; the C-link: 3.6 Å with 249,414 particles). To resolve structures (A-/B-/C-microtubule regions) associated with the proximal A-C linker, particles for the consensus A-C linker were reextracted, where centers were shifted accordingly to individual regions (fig. S4).

For the C-microtubule region, a total of 155,485 particles was extracted with a pixel size of 1.34 Å per pixel and a box size of 360 pixels. One round of 3D classification was performed with a segmented portion of *Tetrahymena* MTT map (EMD-42776) (*12*) as the initial model, resulting in one 3D class associated with the B-C junction that was then used as a reference for downstream processing. All particles were locally refined against the updated reference, followed by Bayesian polishing, resulting in a 4.3-Å resolution reconstruction of the B-C junction. The consensus B-C junction map was further segmented into two subregions (focusing on POC1 and WD40 regions), and focused refinements on these subregions were performed as described above. A total of 231,875 particles was used to determine a 3.8-Å resolution reconstruction of the POC1 region, while a 4.0-Å resolution reconstruction of the WD40 region was determined from 124,254 particles.

For the A-/B-microtubule region, similar image processing pipelines were applied as for the C-microtubule region (fig. S4), resulting in a 6.4-Å resolution reconstruction of the A-microtubule from 41,701 particles and a 5.8-Å resolution reconstruction of the A-B junction from 19,787 particles. Additional focused refinement on the A-microtubule was performed, where 136,681 particles were used to determine a 4.1-Å resolution reconstruction focused on the A05-A09 protofilaments.

To generate a composite map for model building, refinement, and deposition, locally refined maps were aligned to consensus refinement maps of the proximal and transitional A-C linker using the fit in map command in UCSF ChimeraX. For presentation and model building, EMReady (*81*), a 3D nested U-net–based framework for improving the interpretability of cryo-EM maps, was used to sharpen the maps.

The 2D class average in Fig. 6E was generated in the following manner. After performing 3D refinement of the proximal A-C linker in RELION, we used the resulting run_data.star file to reextract 89,664 particles with a box size of >100 nm. These particles with the Euler angles from the previous 3D refinement were used directly for 2D classification with the flag –skip_alignment to generate 2D class averages, and a representative average showing a clear view of the transition between the proximal and transitional A-C linker is shown in Fig. 6E.

### Mass spectrometry

Approximately 50 μg of isolated centrioles were denatured in 8 M urea and treated with tris(2-carboxyethyl)phosphine and iodoacetamide to reduce disulfide bonds and alkylate free thiol groups, respectively. Proteins were digested with endoproteinase Lys-C (FUJIFILM Wako; 3 hours at 37°C), followed by trypsin (Promega; overnight at 37°C) at enzyme/substrate ratios of 1:100 and 1:50, respectively. The digest was acidified to inactivate trypsin and purified by solid-phase extraction using Sep-Pak tC18 columns (Waters).

The purified sample was analyzed in duplicate by injecting an estimated 1 μg of peptides for liquid chromatography–tandem MS on an Easy-nLC 1200 LC system coupled to an Orbitrap Fusion Lumos MS instrument (both Thermo Fisher Scientific). Peptides were separated on an Acclaim PepMap RSLC C18 column (250 mm by 75 μm; Thermo Fisher Scientific) with a 90-min gradient from 4 to 32% acetonitrile in water/0.15% formic acid at a flow rate of 300 nl/min. The mass spectrometer was operated in data-dependent acquisition mode with a cycle time of 3 s (top speed mode). Precursor ions and fragment ions were detected in the Orbitrap analyzer at 60,000 and 15,000 nominal resolution, respectively. Peptides were sequenced by higher-energy collision-induced dissociation at a normalized collision energy of 30%.

Proteins were identified by searching against the *Tetrahymena* reference proteome obtained from UniProt (version 2023_04; with 26,975 entries) using Mascot (Matrix Science; version 2.5.1) with the following parameters: enzyme = trypsin, MS1 error tolerance = 10 parts per million (ppm), MS2 error tolerance = 15 ppm, up to two missed cleavages, instrument type = ESI-QUAD, fixed modification = carbamidomethyl (C), and variable modification = oxidation (M). The error rate was controlled at a false discovery rate of 1% at the peptide level using the built-in decoy function of Mascot.

### AlphaFold predictions

A total of 1536 binary protein-protein interactions was selected on the basis of proteins identified in the MS data and manual curation for structural predictions using AlphaFold2 Multimer v2.3.1 (*82*, *83*). To search for homologous sequences, the standard databases and tools as defined in the AlphaFold2 pipeline were used. The generation of multiple sequence alignments was performed with the default values for all parameters. For recycling, we maximally ran 20 steps.

### Model building and protein identification

Initial model building was performed manually in Coot 0.9.8.93 (*84*) and UCSF ChimeraX (*69*). The interpretation of the proximal A-C linker map started with manual tracing of polyalanine α helices in the A-link and C-link parts, where maps were low-pass filtered to 6 Å. For tubulin dimers and MIPs in the A-microtubule, we built the structure by fitting the available atomic model of the *Tetrahymena* axoneme microtubule doublet [Protein Data Bank (PDB) entry: 8G2Z] (*34*) into our SPA maps.

All globular densities, except tubulins and the A-microtubule MIPs, were assigned by unbiased fitting of AlphaFold2-predicted models of MS-detected proteins into A-C linker maps using the COLORES program [Situs package (*39*)] as previously described (*38*). Briefly, models of *Tetrahymena* monomeric proteins were downloaded from the AlphaFold2 database, while focused refined maps were used as the inputs for COLORES fitting. For each region, the matching with all AlphaFold2 models was scored and ranked on the basis of the cross-correlation value, and the top 50 hits were further visually checked in UCSF ChimeraX. Using this method, we found that the doughnut-shaped density in the A-link belongs to the *Tetrahymena* homolog of WRAP73 (UniProt accession: I7MIG5), and the α-helical domain protein in the B-C junction belongs to I7LY02, which lacks homologs outside of Ciliophora.

To assign other proteins (see below), we performed AlphaFold2 Multimer prediction of potential candidates that were detected in our MS result and manually examined high-scoring fits of COLORES-docked models in the focused refined maps, as described above.

POC1 (UniProt accession: Q22Z96) was previously assigned to the B-C junction based on the comparison between STA results of wild-type centriole and POC1 knockout *Tetrahymena* centrioles (*12*). Consistently, COLORES docked the AlphaFold2-predicted model of POC1 and tubulin dimer model [UniProt accession: I7M9N6 (α-tubulin) and Q24B92 (β-tubulin)] to the B-C junction.

TBC1D31 was previously assigned to the human A-C linker based on U-ExM (*6*), and two homologs of human TBC1D31 [UniProt accession: Q23K58 (TBC1D31A) and Q23AD1 (TBC1D31B)] were found in *Tetrahymena*, where TBC1D31A/B was detected at similar protein abundance by comparing the total unique peptide count in the MS result of *Tetrahymena* centrioles. AlphaFold2 Multimer predictions suggested that TBC1D31A/B can form a dimer via their Rab-GAP domain, and the C-terminal region of POC1 can form a potential trimer with the Rab-GAP domains of TBC1D31A/B. COLORES matched the POC1-TBC1D31A/B trimer (residues 346 to 634 of POC1, 394 to 691 of TBC1D31A, and 420 to 705 of TBC1D31B) to the C-link. By tracing the density around the N and C termini of each subunit of this trimer, we further identified that the α helix on the outer surface of C-microtubule belongs to POC1, and the central filament is built by the long C-terminal α helices of TBC1D31A/B.

CEP135 has been characterized as a structural component of centrioles, which was previously proposed to localize to the A-C linker (*50*), triplet base (*47*), and pinhead-cartwheel (*48*, *49*). Two homologs of human CEP135 (UniProt accession: Q22AS4 and Q24GZ2) were found in *Tetrahymena.* Q22AS4 was previously characterized in genetic studies of *Tetrahymena* and has higher homology to human CEP135 compared to Q24GZ2, while Q24GZ2 is a shorter version of CEP135 having a similar N-terminal portion. Previous work suggested that WRAP73 and CEP135 form a complex (*45*, *46*). Our docking of the AlphaFold2 Multimer model of WRAP73 and CEP135 dimer suggested that CEP135 contributes a coiled coil that binds WRAP73, which was further verified on the basis of the unique arrowhead-shaped structure formed by the N-terminal portion of the CEP135 dimer. Because Q22AS4 and Q24GZ2 have very similar structures in their N-terminal portions, we cannot confidently discriminate between these two homologs in the CEP135 density. We therefore assigned the density as Q22AS4, because this homolog was previously characterized as the canonical CEP135 homolog in *Tetrahymena* (*50*).

All chains were combined in UCSF ChimeraX, and all side chains were stripped using the phenix.pdbtools truncation to poly-ALA command in Phenix (*85*). Models were subjected to refinement (A_asymm_refine.xml) in Rosetta (*86*). Two models are listed in table S2: (i) a single repeating unit of the proximal A-C linker (PDB: 9QZC) and (ii) a composite model of the proximal A-C linker containing six repeating units (PDB: 9QZF).

### Ultrastructure expansion microscopy

Modified *Tetrahymena* cells were cultured in 2% SPP medium (2.0% proteose peptone, 0.2% dextrose, 0.1% yeast extract, and 90 μM Fe-EDTA) at 30°C to mid-log phase (250,000 to 500,000 cells/ml), as determined by a Beckman Z1 Coulter Counter. CEP135-Q22AS4 (TTHERM_01164140) and CEP135-Q24GZ2 (TTHERM_0457090) were ligated into overexpression vectors, which use a metallothionein (MTT) promoter to drive expression in the presence of heavy metals. MTT-GFP-CEP135-Q22AS4 was then ligated into a vector targeting the endogenous CEP135 locus, while MTT-GFP-CEP135-Q24GZ2 was ligated into a vector targeting the RPL29 locus, which confers resistance to cycloheximide (*87*). They were each then transformed into B2086.2 wild-type cells by biolistic bombardment. TBC1D31A-GFP and TBC1D31B-GFP were ligated into endogenous locus vectors and transformed into SB1969 cells and IA267 cells, respectively. Linker sequences of NLE and NLEGSSTSLYKKAGSTMGTNSVDWIRYL were added between the end of MTT-GFP and the start of CEP135-Q22AS4 and CEP135-Q24GZ2, respectively. Linker sequences of SKETAAAKFERQHMDSYVLEVLFQGPVQT and EFRSKETAAAKFERQHMDSYVLEVLFQGPVQT were added between the end of TBC1D31A and TBC1D31B, respectively, and the start of GFP. MTT-GFP-CEP135-Q22AS4, TBC1D31A-GFP, and TBC1D31B-GFP were assorted to paromomycin (200 μg/ml) before imaging, while MTT-GFP-CEP135-Q24GZ2 was assorted to cycloheximide (15 μg/ml). MTT-GFP-CEP135-Q22AS4 and MTT-GFP-CEP135-Q24GZ2 were induced with CdCl_2_ (1.0 μg/ml) for 2 hours before fixation.

U-ExM was performed using a modified protocol (*6*, *12*). Mid-log cells were centrifuged at ×500*g* in 1.5-ml Eppendorf tubes and fixed in 1.4% formaldehyde/2% acrylamide solution in phosphate-buffered saline (PBS) for 18 hours at 37°C on a nutator. Following fixation, cells were pelleted. A total of 15 μl of the cell pellet was added to an 18 mm–by–18 mm coverslip and allowed to dry for 20 min. Gelation medium was generated by adding 5 μl 10% N,N,N′,N′-tetramethylethylenediamine (TEMED) and 5 μl 10% ammonium persulfate (APS) to 90 μl monomer solution (19% sodium acrylate, 10% acrylamide, and 0.1% bisacrylamide in PBS) for a final concentration of 0.5%. Immediately following addition of TEMED and APS, 50 μl of gelation medium was added to a humidity chamber on ice. A coverslip with partially dried cells was then added face down to the gelation solution and incubated for 5 min on ice, followed by 1 hour at 37°C. Five millimeters of circular gel pieces were cut from the solidified gel using a punch and were incubated in 1.5-ml denaturation buffer [500 μM SDS, 200 mM NaCl, and 50 mM tris (pH 9.0)] at 95°C for 1 hour and 30 min. Gels were then washed 2× for 30 min in a 50-ml conical with ddH_2_O, followed by a third ddH_2_O overnight wash.

For immunostaining, gels were washed 2× for 15 min in PBS and incubated in primary antibodies diluted in PBS-2% BSA for 2 hours at 37°C with agitation. The primary antibodies used were mouse anti–acetylated tubulin (Cell Signaling Technology; 1:500) and rabbit anti-GFP (Torrey Pines Scientific; 1:200). Gels were then washed 3× with PBS–Tween 20 0.1% (PBS-T) for 10 min before incubation in secondary antibodies diluted in PBS with 2% BSA for 2 hours at 37°C. The secondary antibodies used were Alexa Fluor 488 donkey anti-rabbit (Thermo Fisher Scientific) and Alexa Fluor 594 goat anti-mouse (Thermo Fisher Scientific; 1:500). Following secondary incubation, gels were washed 3× with PBS-T and then washed 2× for 30 min in ddH_2_O and incubated in ddH_2_O overnight.

Structured illumination microscopy (SIM) was performed using a Nikon 3D SIM system (Ti2 Eclipse) equipped with a 100× total internal reflection fluorescence objective (numerical aperture: 1.45). Images were captured with a complementary metal-oxide semiconductor camera (Orca-Flash 4.0, Hamamatsu) with a *z*-step size of 400 nm. Raw SIM images were reconstructed by the Nikon Elements image stack reconstruction algorithm (Nikon Elements).

Protein localization was performed using a previously developed analysis pipeline (*6*). Briefly, small stack sizes of two to four planes were maximum projected using Fiji (*88*), and the longitudinal view of the centriole was oriented so that the distal end faced the top of the image. Images were then resized, and line scans were generated from lines encompassing the entire centriole. For each centriole measured, the total distance of the acetylated tubulin signal and GFP-tagged protein signal was determined as the width of signal at 50% peak fluorescence intensity using the “PickCentrioleDim” Fiji macro (*6*). The position of the acetylated tubulin signal was shifted and set as 0, and the GFP protein signals were shifted by the same amount to determine their relative position within the centriole. Their coverage was then determined and expressed as a percentage of the acetylated tubulin signal. The length of each centriole was then corrected for gel expansion, and a graph was generated displaying the coverage of each protein relative to acetylated tubulin using the “CentrioleGraph” Fiji macro (*6*).
