# Supplementary material for: Structure and assembly of the A-C linker connecting microtubule triplets in centrioles
Source: Sci Adv. 2025 Oct 8;11(41):eady3689. doi: 10.1126/sciadv.ady3689 (PMC12506977; doi:10.1126/sciadv.ady3689)
Supplement: Supplementary file 1 — Figs. S1 to S15 Tables S1 to S4 Legends for tables S5 to S8 Legends for movies S1 and S2 [file sciadv.ady3689_sm.pdf]

Science Advances
AAAS

Supplementary Materials for

Structure and assembly of the A-C linker connecting microtubule triplets in centrioles

Bin Cai *et al.*

Corresponding author: Michal Wieczorek, michal.wieczorek@mol.biol.ethz.ch

Sci. Adv. **11**, eady3689 (2025)
DOI: 10.1126/sciadv.ady3689

The PDF file includes:

Figs. S1 to S15
Tables S1 to S4
Legends for tables S5 to S8
Legends for movies S1 and S2

Other Supplementary Material for this manuscript includes the following:

Tables S5 to S8
Movies S1 and S2

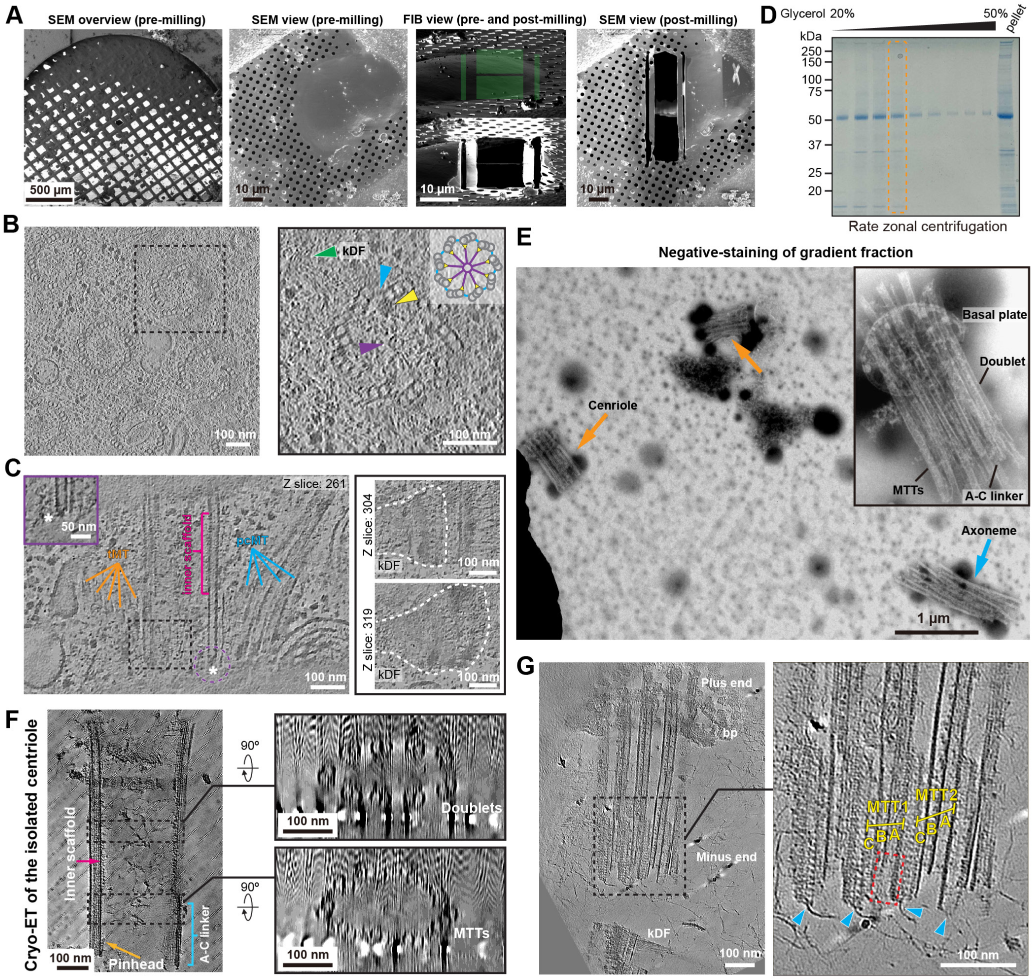

Fig. S1. Cryo-ET analysis of centrioles in FIB-milled *Tetrahymena* cells and in isolated material. (A) Cryo-FIB milling pipeline of *Tetrahymena*. Shown is one example for the preparation of a lamella from a *Tetrahymena* cell. The EM grid with plunge-frozen cells was imaged using a cryo-scanning electron microscopy (SEM overview, pre-milling). The target was identified in SEM view (SEM view, pre-milling), which was further imaged using focused ion beam (FIB) from a shallow angle (FIB view, pre-milling). A milling pattern (green box) was set up and materials was then removed via FIB. The final lamella was inspected using FIB (FIB view, post-milling) and SEM (SEM view, post-milling). **(B)** Slice through a representative cryo-tomogram of a FIB-milled *Tetrahymena* cell (1.8 nm projection), showing discernible centriolar components. A centriole is highlighted by a dashed box and the zoom-in is shown on the right. Several centriole components (the cartwheel: purple; the pinhead: yellow; and the A-C linker: blue) and the kinetodesmal fiber (kDF) are indicated with arrowheads. A cross-sectional schematic of the minus end region of the centriole is shown on the top-right, with centriolar modules colored accordingly. **(C)** Slice through a transverse view of a centriole from a cryo-tomogram of a FIB-milled *Tetrahymena* cell (1.8 nm projection), showing the complex cytoskeleton network associated with the centriole. The centriole core, inner scaffold, transverse microtubules (tMT), and post-ciliary microtubules (pcMT) are labeled. The proximal end of one microtubule capped by γ-TuRC-like structure is highlighted by an asterisk, where the

local region is dashed circled and the zoom-in is shown on the top-left. The contact regions between centriole and kinetodesmal fiber (kDF) are highlighted by a dashed boxed, with views at different Z-height shown on the right. The area occupied by the kinetodesmal fiber is indicated by white dashed lines. **(D)** Coomassie-stained SDS-PAGE gel of rate zonal centrifugation fractions obtained during centriole isolation. The fraction applied for the downstream imaging is highlighted by a dashed orange box. **(E)** Representative negative stain EM image of isolated centrioles (orange arrows). An axoneme contamination is marked by blue arrow. Inset shows a zoomed-in view of an isolated centriole, with different centriolar subcompartments labeled. **(F)** Slice through a cryo-tomogram (same as shown in Fig. 1F but at a different Z-height; 1.1 nm projection) of an isolated centriole. The inner scaffold and pinhead are indicated. Zones occupied by MTTs and microtubule doublets are highlighted by dashed boxes, with corresponding cross-sectional views shown on the right. **(G)** Slice through a cryo-tomogram of an isolated centriole (1.1 nm projection), showing the A-C linker associated central filament emanating from the minus end of the centriole. The kinetodesmal fiber (kDF), basal plate (bp), and minus end, central and plus end portions of the centriole are indicated. The minus end region of the centriole is highlighted by a dashed box and the zoom-in is shown on the right. A-/B-/C-microtubules in two MTTs (MTT1 and MTT2) are labeled. A-C linker-associated central filaments are marked by blue arrowheads. Note that the minus end of the A-microtubules (one highlighted by a red dashed box) capped by a γ-TuRC-like structure *in situ* (Fig. S1C) is lost upon isolation.

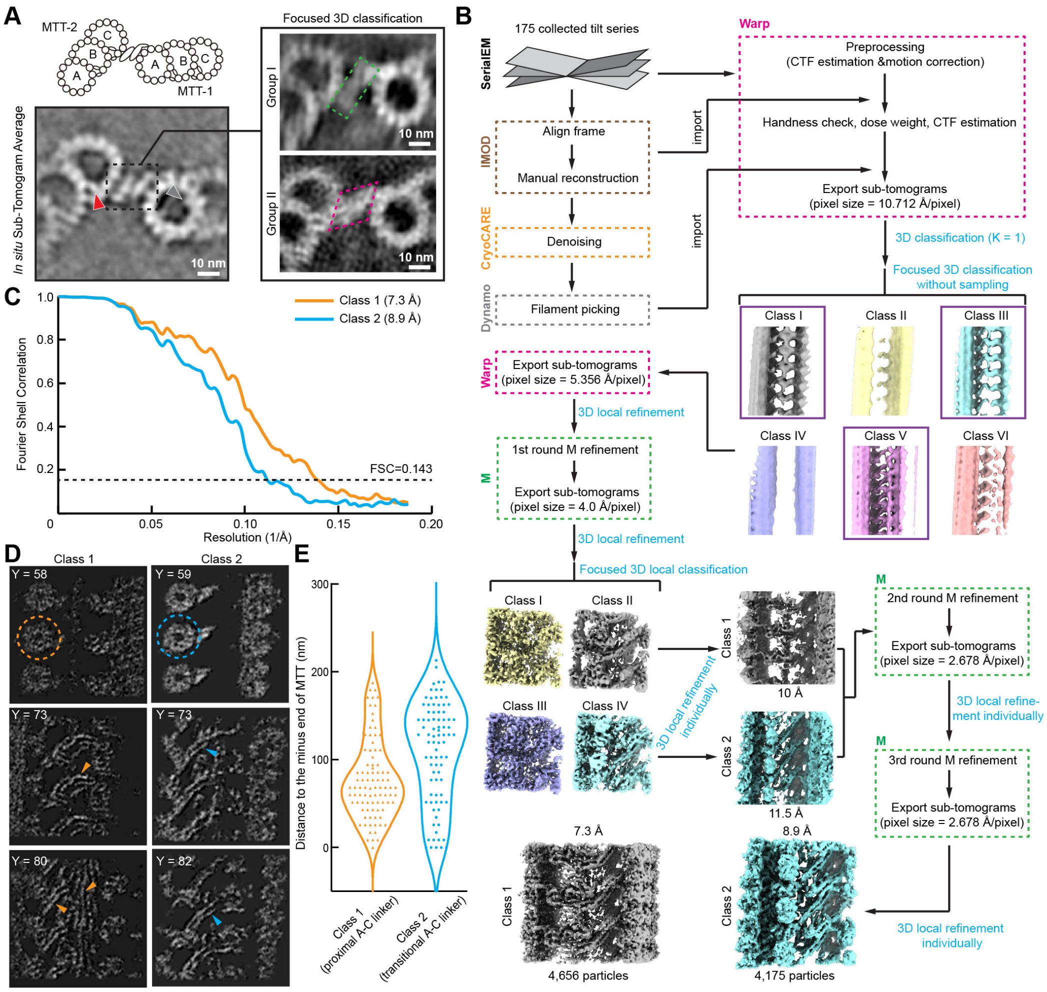

Fig. S2. STA analysis of the A-C linker. (A) Slice through the STA reconstruction result of the *in situ* A-C linker from FIB-milled *Tetrahymena* cells (0.9 nm projection), with a scheme of the A-C linker connecting two MTTs shown on the top. Note that the B-C junction (red arrowhead) and microtubule inner proteins (MIPs, gray arrowhead) are observable in the averaged volume. The A-C linker is marked by a dashed box. Further focused 3D classification on the A-C linker revealed two classes (Groups I and II). Slices at the same Z-height from the two 3D classes are shown on the right, and the A-C linker is indicated by colored dashed boxes (Group I: green, Group II: magenta). **(B)** Flowchart for the STA processing pipeline of the A-C linker in isolated centrioles. See Methods for details. **(C)** Gold-standard Fourier Shell Correlation (FSC) plots of sub-tomogram averages of two A-C linker classes (Class 1: orange, Class 2: blue). **(D)** Transverse views of two 3D classes (Class 1: orange, Class 2: blue) of the A-C linker at different Y-height, showing that some secondary structural features (α-helices: arrowheads) and a similar doughnut-shaped density (dashed circles) are clearly visualized in both reconstruction maps. **(E)** Plot of the relative distance to the proximal end of MTTs from particles used in STA, showing that particles from Class 1 are located much closer to the proximal end of MTTs than particles from Class 2 (Class 1: 77.1 ± 45.3 nm, Class 2: 109.3 ± 57.9 nm, mean ± SD).

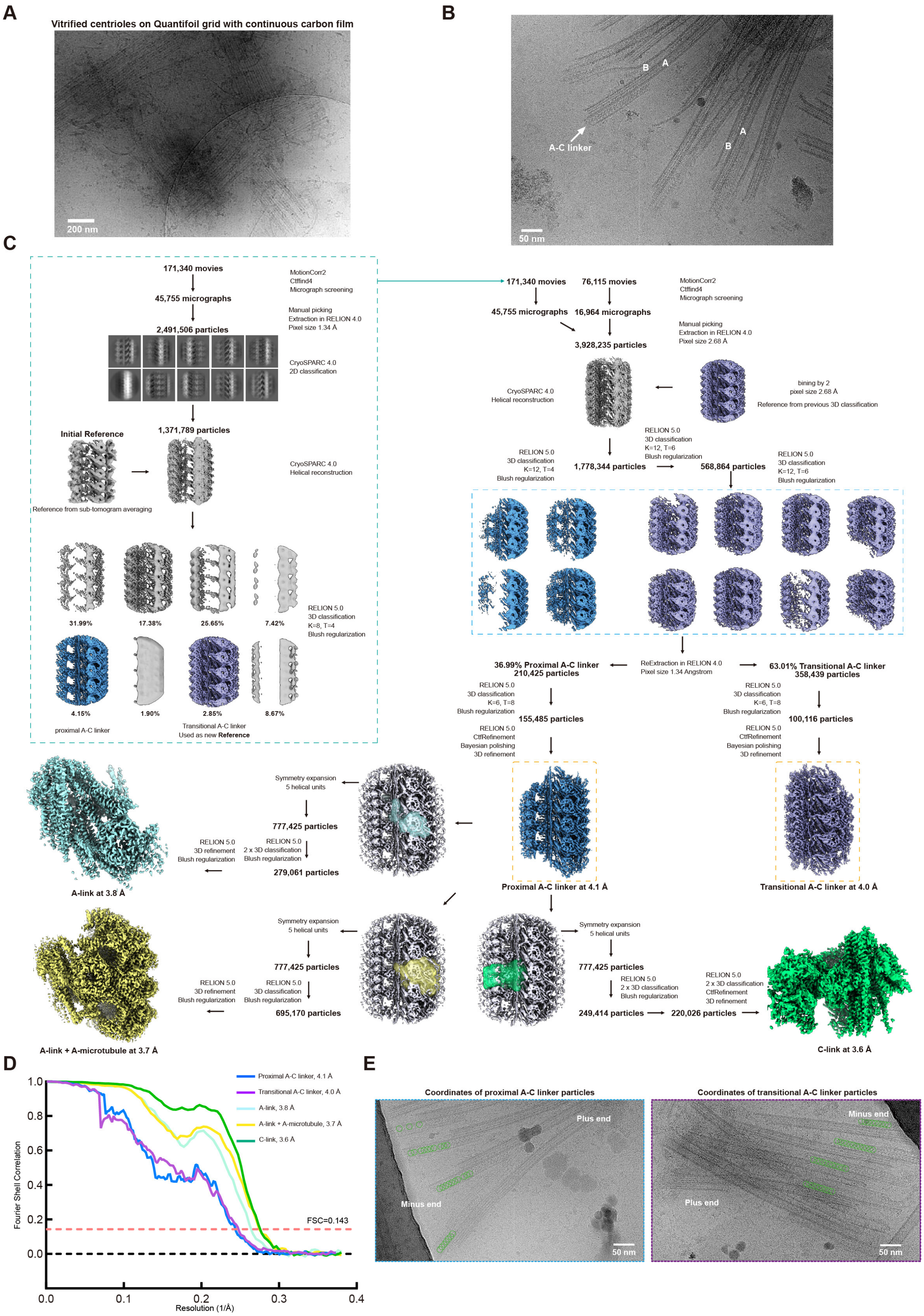

A
Vitrified centrioles on Quantifoil grid with continuous carbon film
200 nm
B
B
A
A-C linker
A
B
50 nm
C
171,340 movies
MotionCorr2
Ctffind4
Micrograph screening
45,755 micrographs
Manual picking
Extraction in RELION 4.0
Pixel size 1.34 Å
2,491,506 particles
CryoSPARC 4.0
2D classification
1,371,789 particles
Initial Reference
Reference from sub-tomogram averaging
CryoSPARC 4.0
Helical reconstruction
31.99%
17.38%
25.65%
7.42%
RELION 5.0
3D classification
K=8, T=4
Blush regularization
4.15%
1.90%
2.85%
8.67%
proximal A-C linker
Transitional A-C linker
Used as new Reference
171,340 movies
76,115 movies
MotionCorr2
Ctffind4
Micrograph screening
45,755 micrographs
16,964 micrographs
Manual picking
Extraction in RELION 4.0
Pixel size 2.68 Å
3,928,235 particles
CryoSPARC 4.0
Helical reconstruction
bining by 2
pixel size 2.68 Å
Reference from previous 3D classification
RELION 5.0
3D classification
K=12, T=4
Blush regularization
1,778,344 particles
RELION 5.0
3D classification
K=12, T=6
Blush regularization
568,864 particles
RELION 5.0
3D classification
K=12, T=6
Blush regularization
36.99% Proximal A-C linker
210,425 particles
ReExtraction in RELION 4.0
Pixel size 1.34 Angstrom
63.01% Transitional A-C linker
358,439 particles
RELION 5.0
3D classification
K=6, T=8
Blush regularization
155,485 particles
RELION 5.0
3D classification
K=6, T=8
Blush regularization
100,116 particles
RELION 5.0
CtfRefinement
Bayesian polishing
3D refinement
RELION 5.0
CtfRefinement
Bayesian polishing
3D refinement
Proximal A-C linker at 4.1 Å
Transitional A-C linker at 4.0 Å
Symmetry expansion
5 helical units
777,425 particles
RELION 5.0
2 x 3D classification
Blush regularization
279,061 particles
RELION 5.0
3D refinement
Blush regularization
A-link at 3.8 Å
Symmetry expansion
5 helical units
777,425 particles
RELION 5.0
3D classification
Blush regularization
695,170 particles
RELION 5.0
3D refinement
Blush regularization
A-link + A-microtubule at 3.7 Å
Symmetry expansion
5 helical units
777,425 particles
RELION 5.0
2 x 3D classification
Blush regularization
249,414 particles
220,026 particles
RELION 5.0
2 x 3D classification
CtfRefinement
3D refinement
C-link at 3.6 Å
D
Proximal A-C linker, 4.1 Å
Transitional A-C linker, 4.0 Å
A-link, 3.8 Å
A-link + A-microtubule, 3.7 Å
C-link, 3.6 Å
FSC=0.143
Fourier Shell Correlation
Resolution (1/Å)
1.0
0.8
0.6
0.4
0.2
0.0
0.0
0.1
0.2
0.3
0.4
E
Coordinates of proximal A-C linker particles
Plus end
Minus end
50 nm
Coordinates of transitional A-C linker particles
Minus end
Plus end
50 nm

Fig. S3. SPA of consensus A-C linker reconstructions. (A-B), Cryo-EM micrographs of isolated centrioles before (**A**; lower magnification) and after (**B**; higher magnification) mechanical disruption to splay centrioles into MTT subassemblies. **(C)** Cryo-EM data processing workflow for consensus reconstructions of both the proximal and transitional A-C linkers, as well as locally processed maps for the proximal A-C linker (A-link, A-link + A-microtubule, and C-link). See Methods for details. **(D)** Gold-standard FSC curves of cryo-EM density maps in **C**. **(E)** Particle coordinates (green circles) from proximal (left) and transitional (right) consensus A-C linker reconstructions mapped back onto representative cryo-EM micrographs.

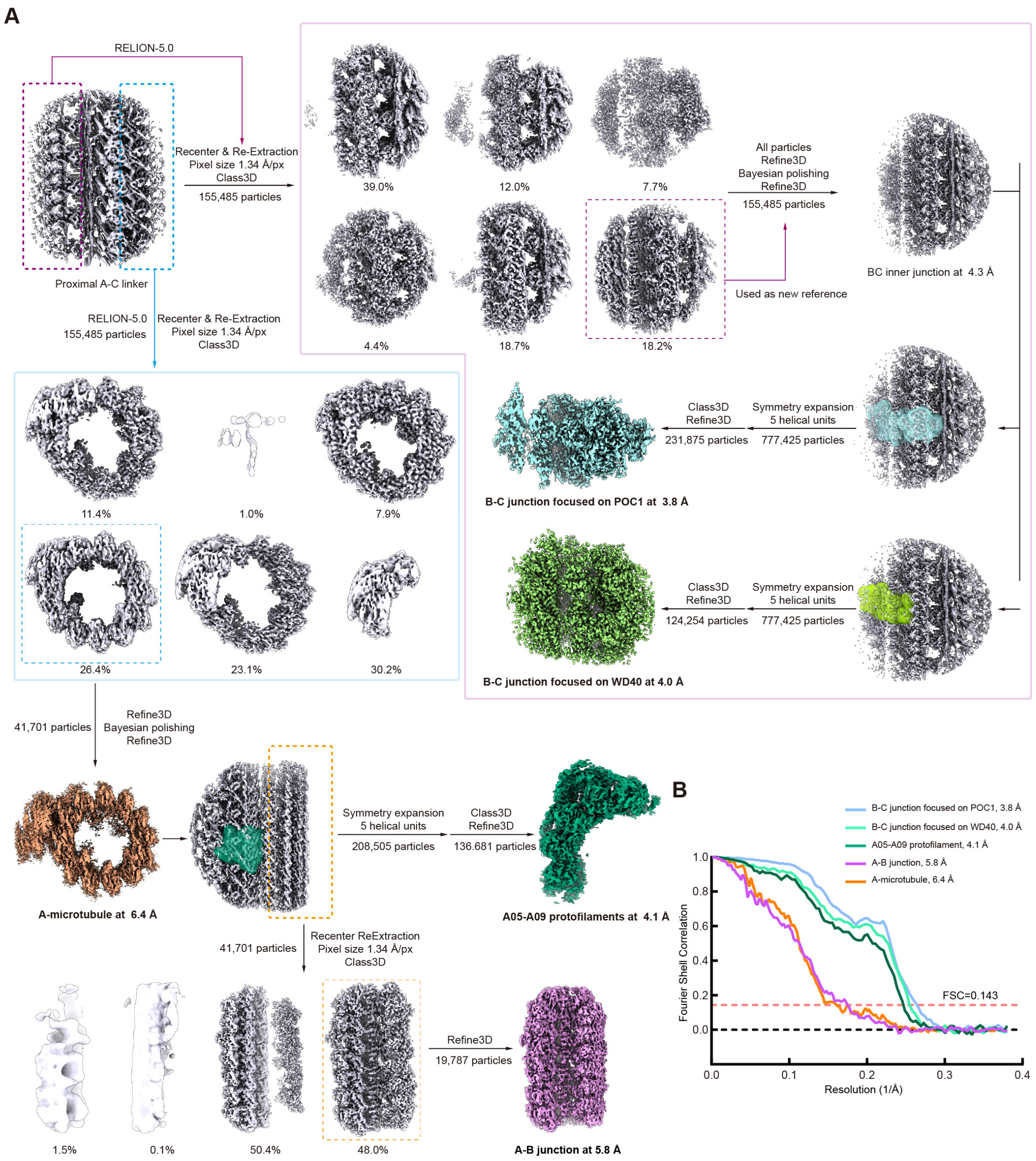

Fig. S4. SPA of proximal A-C linker subregions. (A) Local cryo-EM data processing workflow stemming from the proximal (Class 1) consensus A-C linker reconstruction in Extended Data Figure 3 resulting in the following final reconstructed maps: the B-C junction focused on POC1; the B-C junction focused on WD40; protofilaments A05-A09 of the A-microtubule; the A-B junction; and the A-microtubule. See Methods for details. **(B)** Gold-standard FSC curves of cryo-EM density maps in **A**.

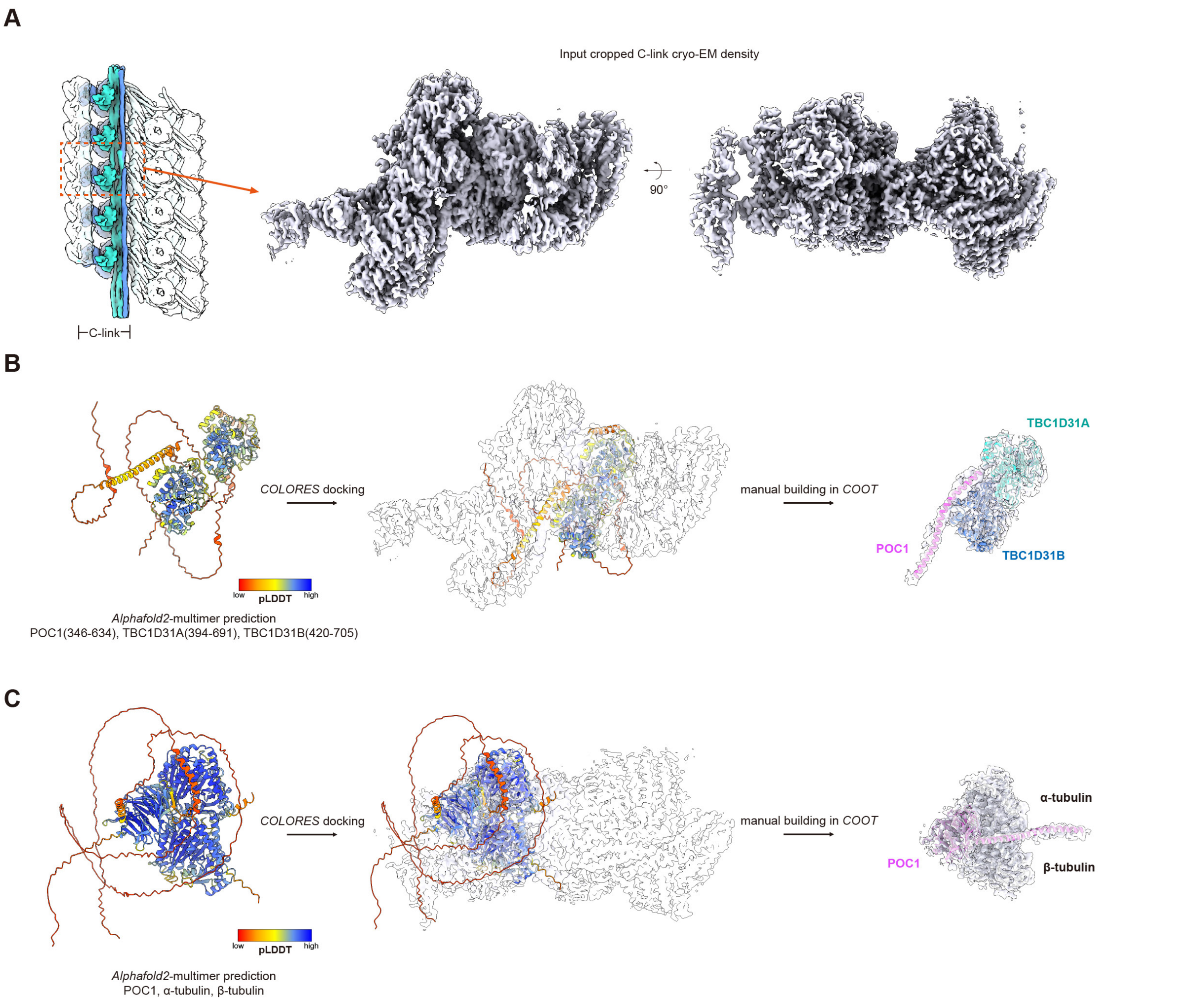

Fig. S5. TBC1D31A/B and POC1 assignment methodology. (A) Two views of the C-link cryo-EM density map. A schematic of the A-C linker with the C-link colored is shown on the left for reference. **(B-C),** AlphaFold Multimer predicted models of POC1 & TBC1D31A/B (**B**) and POC1 & α/β-tubulin (**C**), colored according to predicted local distance difference test (pLDDT) scores and docked (highest scoring docking shown) into the C-link cryo-EM density map by COLORES (*37*). Final adjusted and refined models are shown on the right in cartoon representation, colored according to the legend in Figure 2d, and with their respective cryo-EM densities as white transparent surfaces.

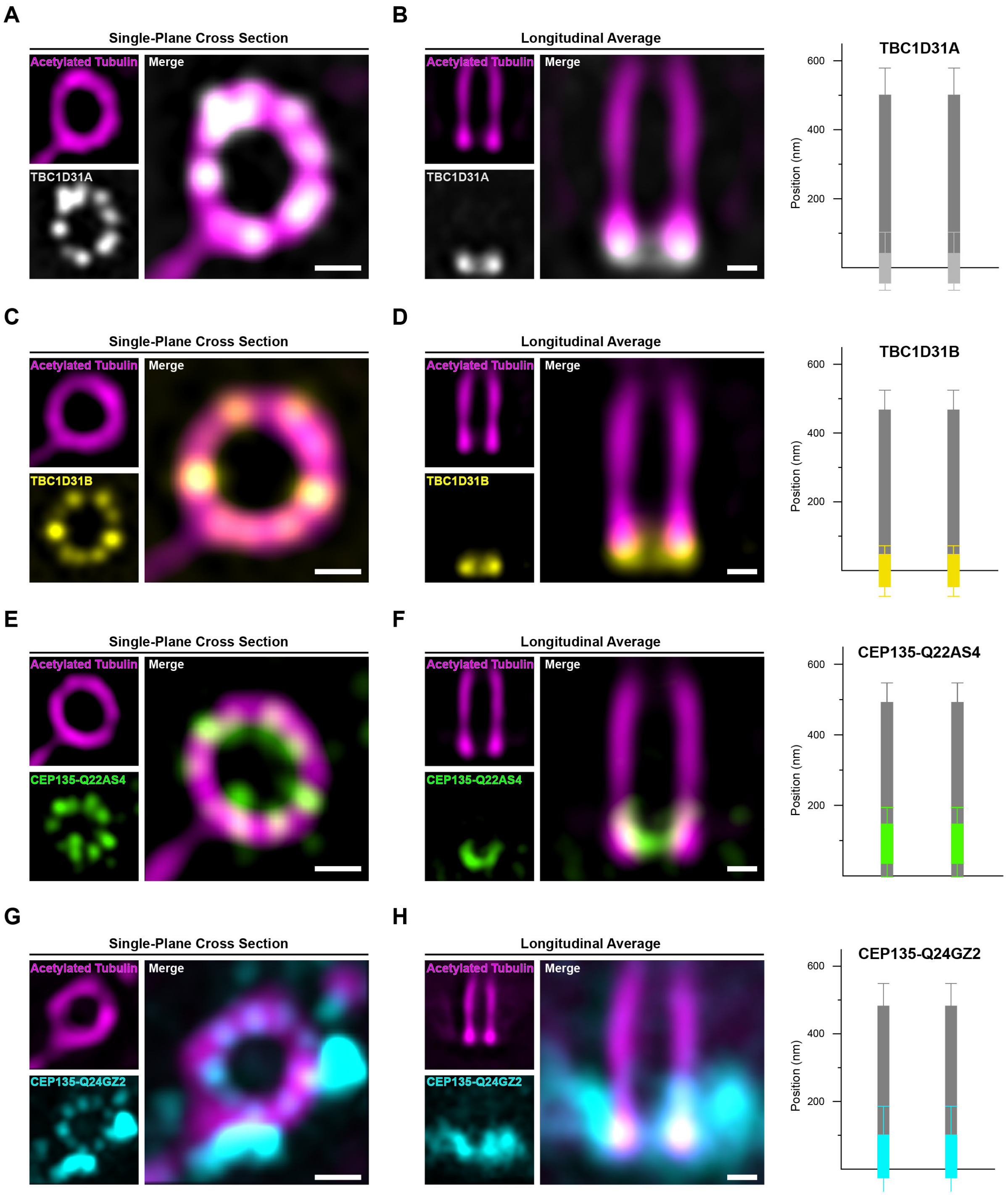

Fig. S6. Expansion microscopy of TBC1D31A/B and CEP135 variants. (A) Representative single-plane basal body cross section showing TBC1D31A (grey) localization, acetylated tubulin (magenta). **(B)** Quantification of average longitudinal position of TBC1D31A in basal body, N=77 basal bodies. (**C**) Representative single-plane basal body cross section showing TBC1D31B (yellow) localization, acetylated tubulin (magenta). **(D)** Quantification of average longitudinal position of TBC1D31B in basal body, N=44 basal bodies. **(E)** Representative single-plane basal body cross section showing CEP135-Q22AS4 (green) localization, acetylated tubulin (magenta). **(F)** Quantification of average longitudinal

position of CEP135-Q22AS4 in basal body, N=64 basal bodies. **(G)** Representative single-plane basal body cross section showing CEP135-Q24GZ2 (cyan) localization, acetylated tubulin (magenta). **(H)** Quantification of average longitudinal position of CEP135-Q24GZ2 in basal body, N=33 basal bodies. Scale bars, 100 nm.

A

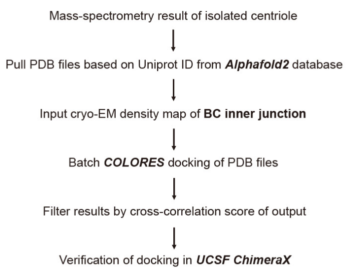

B

Alphafold2 file	Output file name	CC-Value	Protein description
Q24B92	col_best_001.pdb	0.027945	Tubulin beta chain
I7M9N6	col_best_001.pdb	0.027567	Tubulin alpha chain
I7M9N6	col_best_002.pdb	0.026296	Tubulin alpha chain
Q24B92	col_best_002.pdb	0.02555	Tubulin beta chain
I7M9N6	col_best_003.pdb	0.025307	Tubulin alpha chain
Q24B92	col_best_003.pdb	0.025165	Tubulin beta chain
Q22ED2	col_best_001.pdb	0.024785	Tubulin beta chain
Q22ED2	col_best_002.pdb	0.024746	Tubulin beta chain
I7M9N6	col_best_004.pdb	0.024117	Tubulin alpha chain
I7M9N6	col_best_005.pdb	0.023992	Tubulin alpha chain
Q24B92	col_best_004.pdb	0.023856	Tubulin beta chain
Q22ED2	col_best_003.pdb	0.0237	Tubulin beta chain
Q24B92	col_best_005.pdb	0.023639	Tubulin beta chain
Q22ED2	col_best_004.pdb	0.023582	Tubulin beta chain
Q22ED2	col_best_005.pdb	0.023532	Tubulin beta chain
Q24B92	col_best_006.pdb	0.023525	Tubulin beta chain
I7M9N6	col_best_006.pdb	0.023358	Tubulin alpha chain
Q22ED2	col_best_006.pdb	0.022961	Tubulin beta chain
I7M9N6	col_best_007.pdb	0.022896	Tubulin alpha chain
Q24B92	col_best_007.pdb	0.022324	Tubulin beta chain
Q24B92	col_best_008.pdb	0.021929	Tubulin beta chain
Q24D42	col_best_001.pdb	0.021844	Receptor of activated kinase C 1A, component of 40S small ribosomal subunit (ribosomal)
Q24D42	col_best_002.pdb	0.02165	Receptor of activated kinase C 1A, component of 40S small ribosomal subunit (ribosomal)
I7M9N6	col_best_008.pdb	0.021502	Tubulin alpha chain
I7M9N6	col_best_009.pdb	0.021421	Tubulin alpha chain
Q22ED2	col_best_007.pdb	0.021308	Tubulin beta chain
I7LY02	col_best_001.pdb	0.02112	SWIM-type domain-containing protein
W7XF00	col_best_001.pdb	0.020962	Transmembrane protein, putative (mitocondiral)
Q24B92	col_best_009.pdb	0.020218	Tubulin beta chain
Q22ED2	col_best_008.pdb	0.020176	Tubulin beta chain
I7M0B6	col_best_001.pdb	0.019946	WD domain, G-beta repeat protein
Q24D42	col_best_003.pdb	0.019913	Receptor of activated kinase C 1A, component of 40S small ribosomal subunit (ribosomal)
I7M0B6	col_best_002.pdb	0.019826	WD domain, G-beta repeat protein
I7LUQ4	col_best_001.pdb	0.019796	Uncharacterized protein (mitochondrial)
Q24D42	col_best_004.pdb	0.019726	Receptor of activated kinase C 1A, component of 40S small ribosomal subunit (ribosomal)

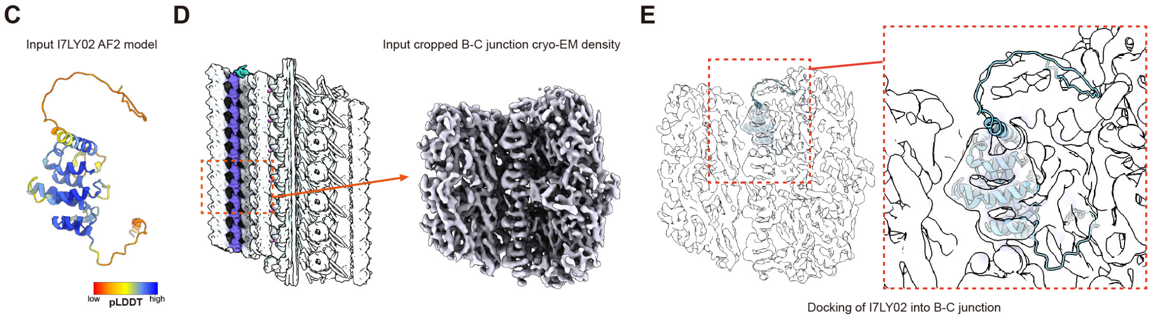

Fig. S7. I7LY02 assignment methodology. (A) Workflow for assignment of proteins to the B-C junction. See Methods for details. **(B)** Ranked output of AlphaFold predicted models docked into the B-C junction densities. One of the non-tubulin top scoring candidates that fit well into the α-helical ladder density, I7LY02, is highlighted. **(C)** AlphaFold model of I7LY02 in cartoon representation and colored according to pLDDT score. **(D)** Segmented B-C junction cryo-EM density map used to score AlphaFold model docking with COLORES(*39*). The schematic of the A-C linker and B-C junction with the I7LY02 ladder density colored is shown on the left. **(E)** Zoomed-in view (right) of the COLORES docking output for I7LY02 (blue cartoon representation) in the segmented density map (transparent surface).

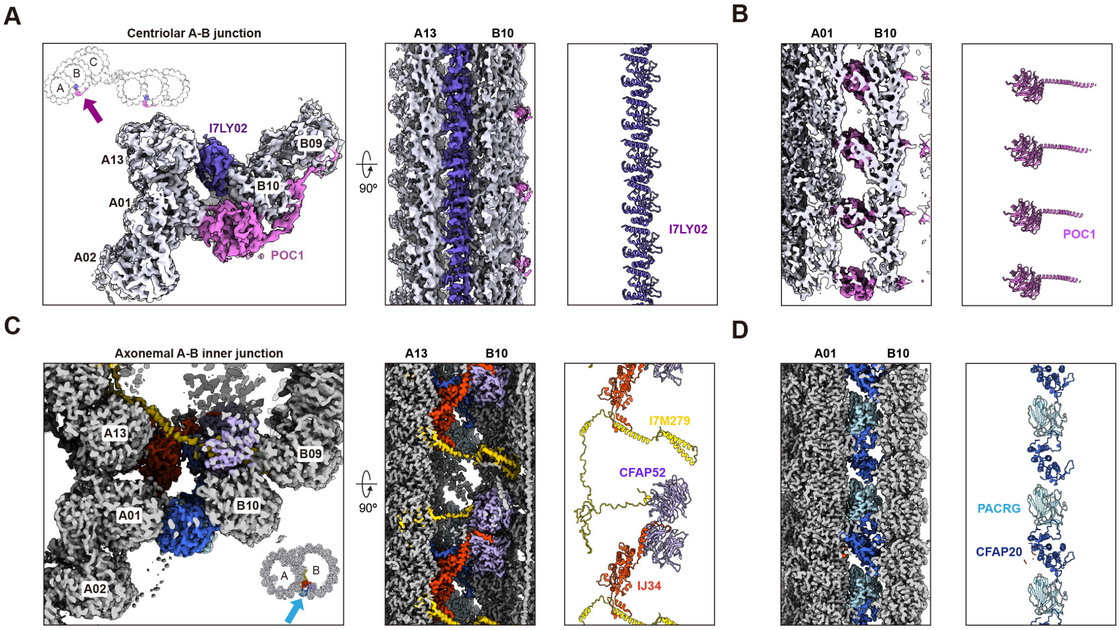

Fig. S8. Comparison of *Tetrahymena* A-B junctions in MTTs and in microtubule doublets. (A) Left: Cross-section view of the centriolar MTT A-B junction density map. POC1, I7LY02, and A- and B-microtubule protofilaments are labeled. A schematic is shown on the top left. Transverse views of the I7LY02 ladder density (middle) and model (cartoon representation; right) are also shown. A schematic is shown in the top left. **(B)** Transverse views of POC1 density (left) and model (cartoon representation; right) in the MTT A-B junction. **(C)** Left: Cross-section view of a published axonemal doublet A-B junction density map (EMD-29685)(*34*). A- and B-microtubule protofilaments are labeled. A schematic is shown in the bottom right. Transverse views of the proteins making up the B-microtubule lumenal part of the A-B junction density (middle) and their corresponding models (PDB ID: 8G2Z; cartoon representation; right)(*34*) are also shown. A schematic is shown in the bottom right. **(D)** Transverse views of PACRG/CFAP20 densities (EMD-29685; left)(*34*) and models (PDB ID: 8G2Z; cartoon representation; right)(*34*) in the axoneme lumenal part of the A-B junction.

A

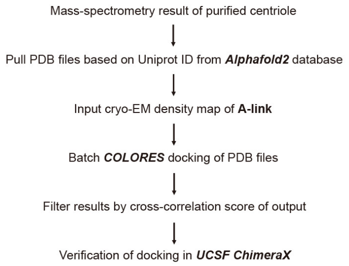

B

Alphafold2 file	Output file name	CC-Value	Protein description
Q22MB0	col_best_001.pdb	0.035914	40S ribosomal protein S29
I7MIG5	col_best_001.pdb	0.035763	WD repeat WRAP73-like protein, putative
I7M1Q5	col_best_001.pdb	0.034872	Transmembrane protein
Q22EC7	col_best_001.pdb	0.034318	Dpy-30 motif protein
I7M1Q5	col_best_002.pdb	0.03348	Transmembrane protein
I7M1Q5	col_best_003.pdb	0.033465	Transmembrane protein
Q22W11	col_best_001.pdb	0.033215	2 iron, 2 sulfur cluster-binding protein
Q22EC7	col_best_002.pdb	0.033121	Dpy-30 motif protein
I7M1Q5	col_best_004.pdb	0.033098	Transmembrane protein
I7MD70	col_best_001.pdb	0.032978	Transmembrane protein
I7M1C3	col_best_001.pdb	0.032606	Xylose isomerase-like TIM barrel domain-containing protein
I7M1N7	col_best_001.pdb	0.032448	Dynein light chain 1, axonemal protein
I7M772	col_best_001.pdb	0.032251	Uncharacterized protein
Q231B2	col_best_001.pdb	0.031924	Uncharacterized protein
Q231C4	col_best_001.pdb	0.031909	Uncharacterized protein
I7MJ34	col_best_001.pdb	0.031893	60S acidic ribosomal protein
I7M294	col_best_001.pdb	0.031862	Uncharacterized protein
Q22W11	col_best_002.pdb	0.031824	2 iron, 2 sulfur cluster-binding protein
Q22NN3	col_best_001.pdb	0.031626	EF-hand domain-containing protein
Q24C99	col_best_001.pdb	0.031575	Kinase domain protein
I7MD70	col_best_002.pdb	0.031482	Transmembrane protein
W7X6G4	col_best_001.pdb	0.031441	Uncharacterized protein
Q237C4	col_best_001.pdb	0.031404	Dpy-30 motif protein
I7MDI8	col_best_001.pdb	0.031367	Bmh1 14-3-3 family protein
I7M2E3	col_best_001.pdb	0.031186	Bmh1 14-3-3 family protein
Q238Q8	col_best_001.pdb	0.03104	40S ribosomal protein S15a
Q238Q8	col_best_002.pdb	0.030997	40S ribosomal protein S15a
Q238Q8	col_best_003.pdb	0.030965	40S ribosomal protein S15a
Q23G22	col_best_001.pdb	0.030963	c-Myc-binding protein, putative
A4VCU8	col_best_001.pdb	0.030895	Flagellar FliJ protein
I7MKQ6	col_best_001.pdb	0.030895	High mobility group (HMG) box protein
Q22W05	col_best_001.pdb	0.030858	Sperm flagellar protein
Q22DQ8	col_best_001.pdb	0.030841	Uncharacterized protein
Q23G98	col_best_001.pdb	0.030828	Large ribosomal subunit protein eL43
Q234E0	col_best_001.pdb	0.030779	Allergen

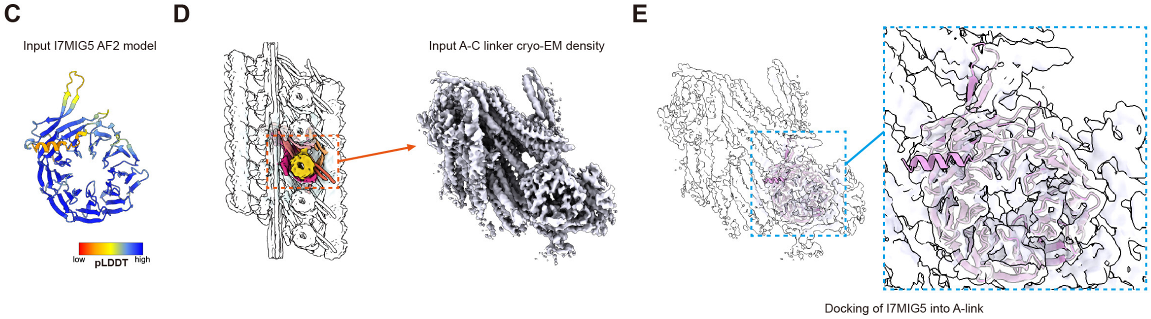

Fig. S9. WRAP73 assignment methodology. (A) Workflow for assignment of WRAP73 to the A-C linker A-link. See Methods for details. **(B)** Ranked output of AlphaFold predicted models docked into the A-link density map. One of the non-tubulin top scoring candidates that fits well into the β-propeller density, WRAP73, is highlighted. **(C)** AlphaFold model of WRAP73 in cartoon representation and colored according to pLDDT score. **(D)** Segmented A-C linker A-link cryo-EM density map used to score AlphaFold model docking with COLORES(*39*). Schematic of the A-C linker with the A-link density colored is show on the left. **(E)** Zoomed-in view (right) of the COLORES docking output for WRAP73 (pink cartoon representation) in the A-link density map (transparent surface).

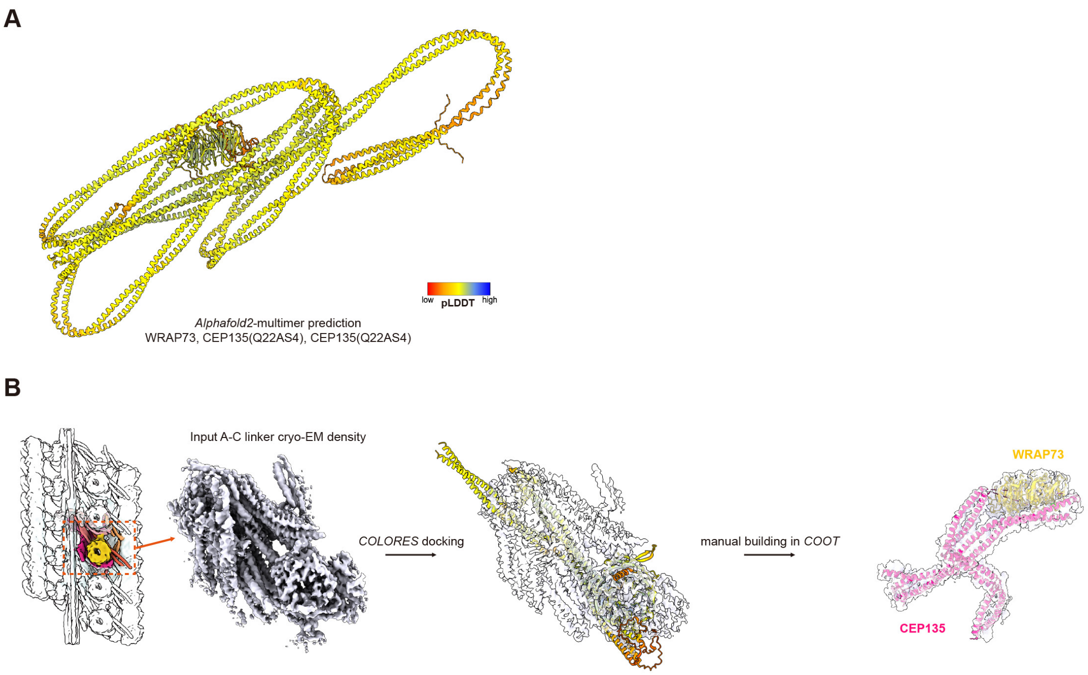

Fig. S10. CEP135 assignment methodology. (A) AlphaFold Multimer predicted models of WRAP73 together with two copies of the “long” CEP135 isoform. The model is shown in cartoon representation and colored according to pLDDT score. **(B)** AlphaFold Multimer predicted model in panel **a** docked by COLORES (middle; highest scoring docking shown) into the A-link cryo-EM density map (left). Final models adjusted and refined models are shown on the right, colored according to the legend in Figure 2d, and with their respective cryo-EM densities as white transparent surfaces. A schematic of the A-C linker is shown on the far left for reference.

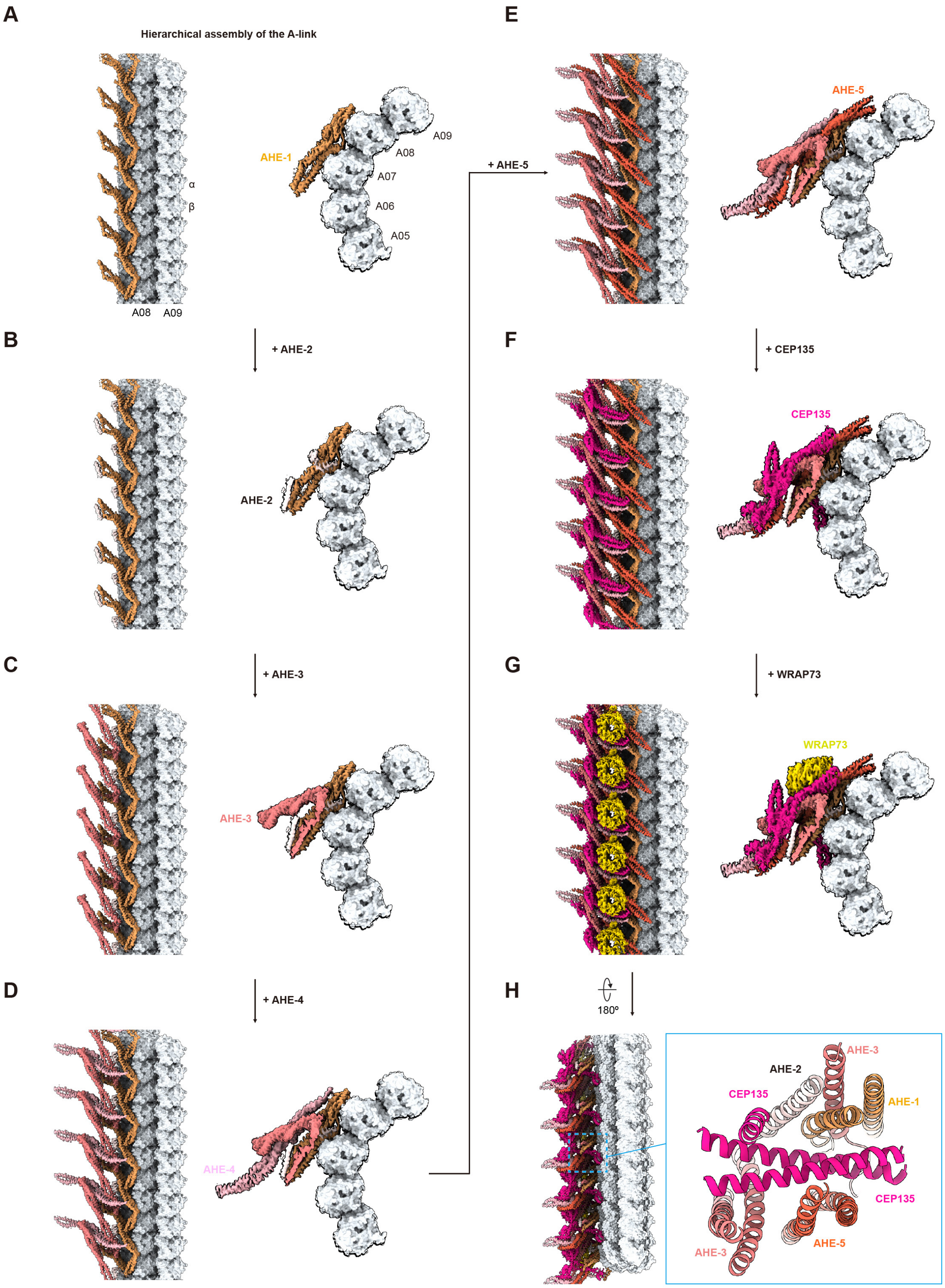

Fig. S11. Hierarchical assembly of the A-C linker A-link. (A-G) Two views (left: transverse view; right: cross-section view) of segmented A-link cryo-EM densities (the proximal A-C linker) showing sequential layering of coiled-coil elements (**A-F**), finalized by the addition of WRAP73 (**G**). **(H)** Transverse view of the A-link showing the organization of the CEP135 dimer with the unassigned coiled-coil elements (AHE1-5).

A zoomed-in view is shown on the right, showing how a dimer of CEP135 residues ~280-320 thread through and organize these elements.

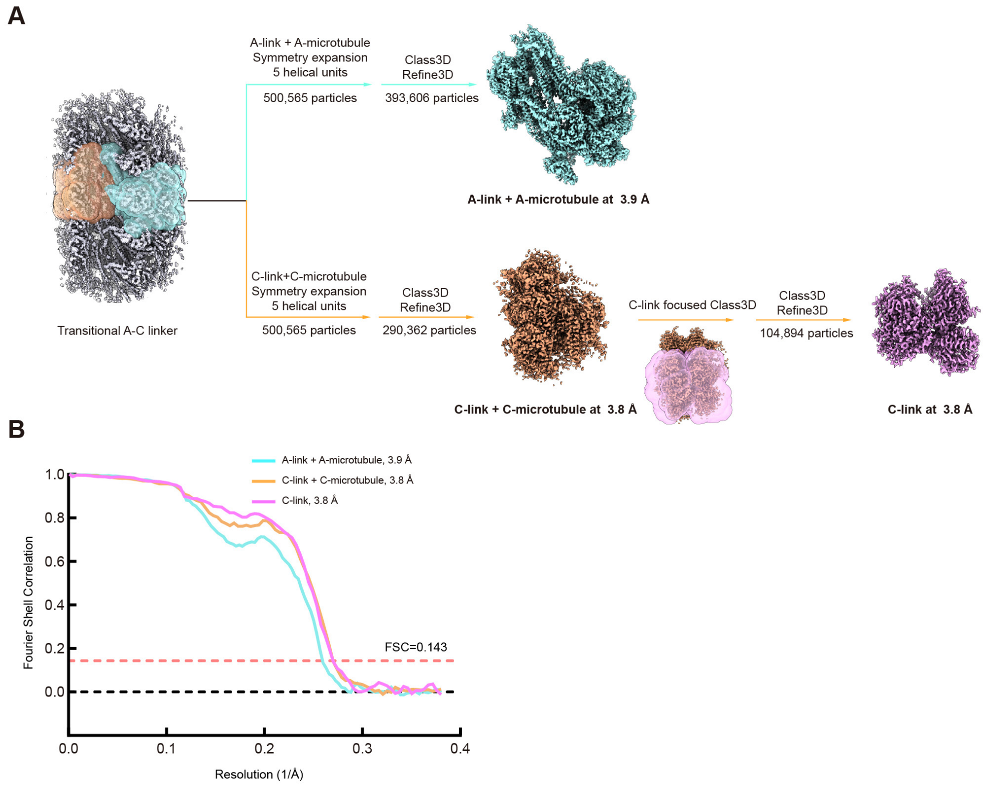

Fig. S12. SPA of transitional A-C linker subregions. (A) Local cryo-EM data processing workflow stemming from the consensus transitional A-C linker reconstruction in Extended Data Figure 3 resulting in the following final reconstructed maps: A-link + A-microtubule; C-link; and C-link + C-microtubule. **(B)** Gold-standard FSC curves of cryo-EM density maps in **A**.

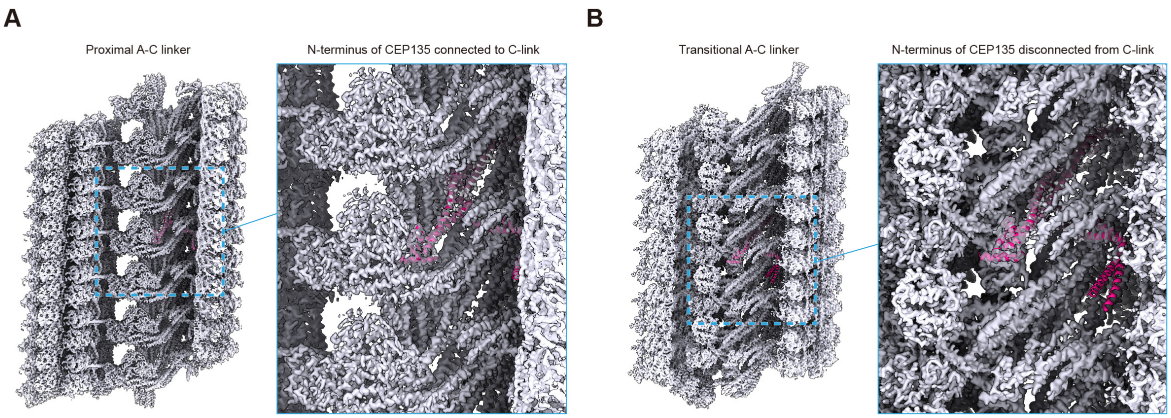

Fig. S13. Comparison of CEP135's N-terminal "arrowhead" structure between the proximal versus transitional A-C linkers. (A-B) Views of the CEP135 model (cartoon representation) in the proximal (**A**) and transitional (**B**) A-C linker composite density maps. Zoomed-in views highlight changes in the densities surrounding CEP135's N-terminus. Model in (**B**) was modified manually using the proximal A-C linker model to fit the altered conformation.

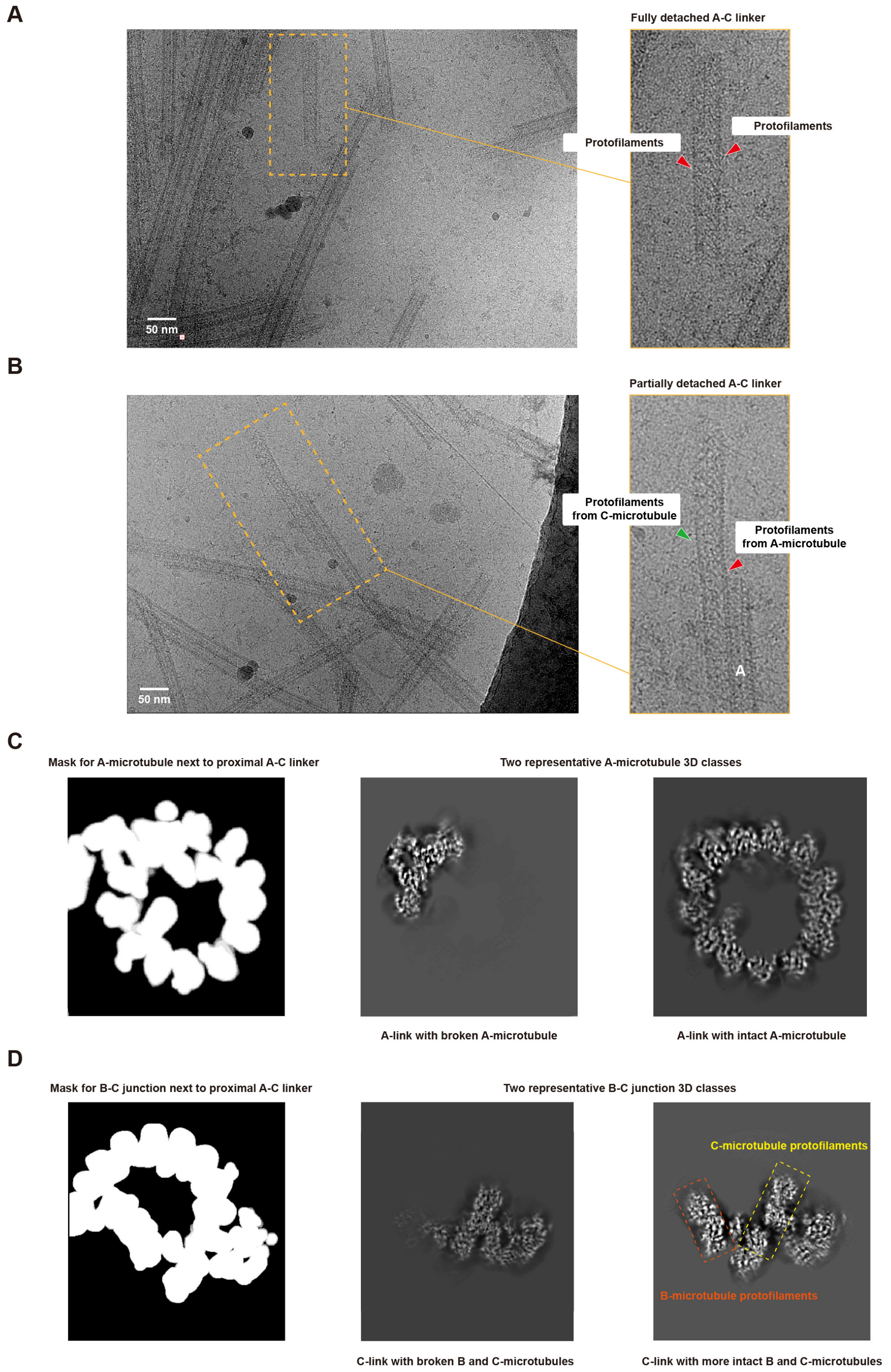

Fig. S14. Dissociated A-C linkers remain bound to tubulin protofilaments. (A-B) Cryo-EM micrographs of mechanically disrupted centrioles. Examples of fully detached (**A**) and partially detached (**B**) A-C linker structures are highlighted by yellow dashed boxes with zoomed-in views on the right. MTT microtubule protofilaments that remain attached to the A-C linker are indicated. **(C-D)** Cross-sectional

views of the A-microtubule (**C**) and the B-C junction density maps (**D**) showing the masks used for 3D classification (left), a 3D classification result showing stripped protofilaments (middle), and a 3D classification result showing more intact microtubules / protofilaments (right). B-, and C- microtubule protofilaments are indicated in (**D**), where relevant.

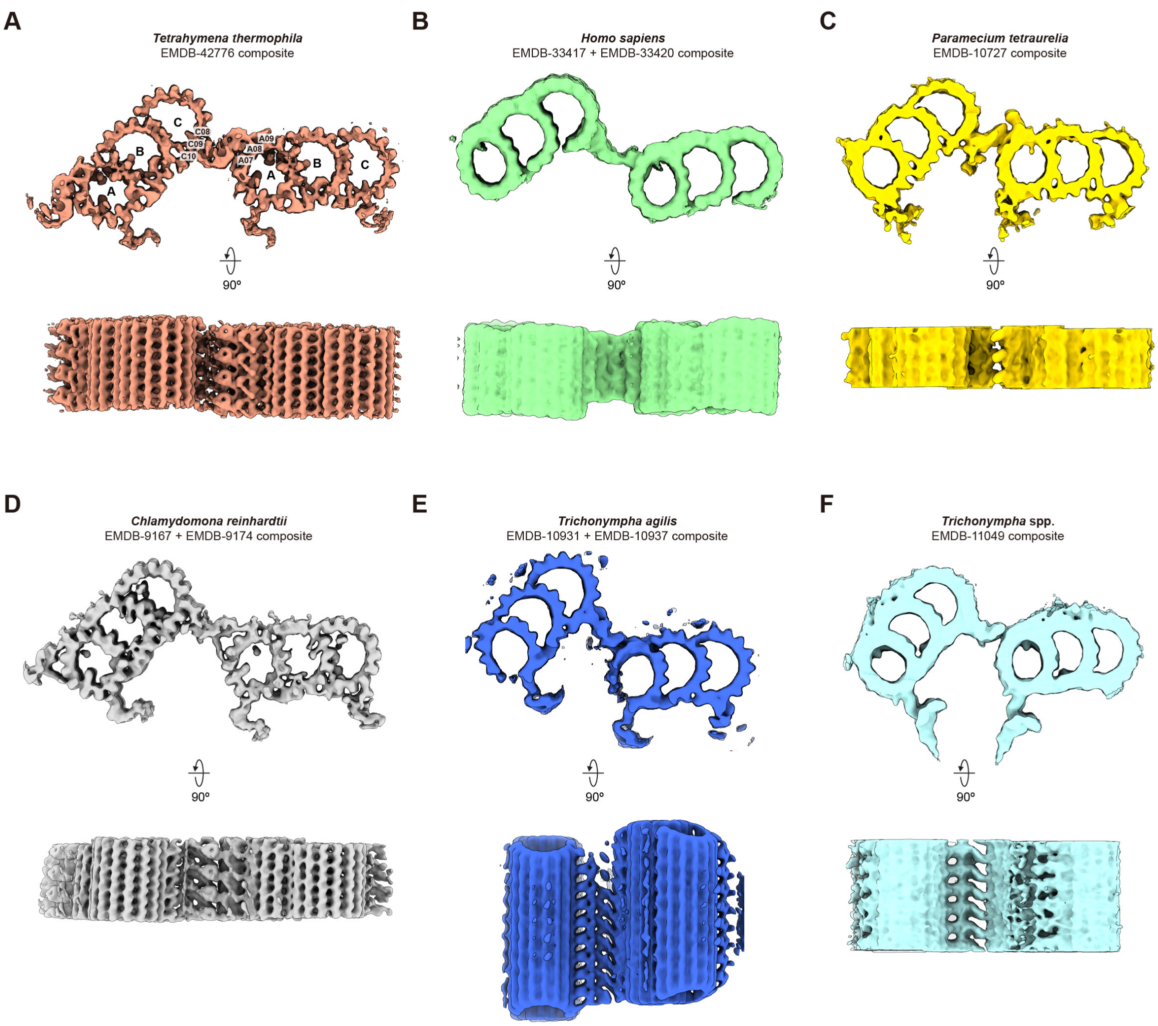

Fig. S15. Comparison of previous A-C linker reconstructions. (A-F) Two views of composite density maps for the A-C linker and two neighboring MTTs from different species previously deposited: *Tetrahymena(13)* (**A**), human*(12)* (**B**), *Paramecium(9)* (**C**), *Chlamydomonas(14)* (**D**), *Trichonympha agilis(15)* (**E**), and *Trichonympha* spp.(*10*) (**F**). The related maps were downloaded from EMDB and composite maps were created in UCSF ChimeraX to generate comparable views of the A-C linker relative to two MTTs.

Table S1. Data collection and refinement statistics of sub-tomogram averaging for the A-C linker.

	Sub-tomogram average of the proximal A-C linker in the isolated centrioles (Class01) (EMDB-54239)	Sub-tomogram average of the transitional A-C linker in the isolated centrioles (Class02) (EMDB-54240)
**Data collection and processing**		
Microscope	FEI Titan Krios G4 TEM	
Camera	Gata K3 with BioContinnum GIF	
Magnification	33,000	
Voltage (kV)	300	
Electron exposure (e^-/Å)	~130	
Total frames	40	
Defocus range (μm)	-3 to -5	
Pixel size (Å/pixel)	2.678	
Symmetry imposed	C1	
Initial particle number	42,510	
**Refinement**		
Map resolution (Å)	7.3	8.9
Final particle number	4,656	4,175
Map sharpening b factor	-109	-57

Table S2. Data collection, refinement and validation statistics of single-particle analysis for the proximal A-C linker.

	Composite map (EMDB-53467) (PDB 9QZC)	Composite map (EMDB-53467) (PDB 9QZF)	A-link map (EMDB-53975)	A-link & A-microtubule map (EMDB-53977)	C-link (EMDB-53978)
**Data collection and processing**					
Microscope			FEI Titan Krios G3i TEM		
Camera			Gata K3 with BioQuantum GIF		
Magnification			64,000		
Voltage (kV)			300		
Electron exposure (e^{-}/Å)			32 - 36		
Total frames			40		
Defocus range (µm)			-0.8 - -3.0		
Pixel size (Å/pixel)			1.34		
Symmetry imposed			C1 and helical		
Initial particle number			3,928,235		
**Refinement**					
Map resolution (Å)	N/A	N/A	3.8	3.7	3.6
Final particle number	N/A	N/A	279,061	695,170	220,026
Map sharpening b factor	N/A	N/A	-134	-130	-105
Model composition					
Atoms	22355	130144			
Protein residues	4492	26153			
Chains	18	114			
R.M.S deviations					
Bond length (Å)	0.018	0.018			
Bond angles (˚)	2.298	2.309			
Validation					
MolProbity score	1.47	1.50			
Clashscore	2.31	2.46			
Rotamer outlier (%)	0.00	0.00			
Ramachandran plot					
Favored (%)	92.70	92.43			
Allowed (%)	5.69	5.85			
Outlier (%)	1.61	1.72			

Table S3. Cryo-EM data collection and refinement statistics for proximal A-C linker associated B-C junction, A-microtubule and A-B junction.

	A-microtubule map (EMDB-53979)	A05-A09 protofilaments (EMDB-53980)	A-B junction (EMDB-53981)	B-C junction POC1 focused (EMDB-53982)	B-C junction WD40 focused (EMDB-53983)
**Data collection and processing**					
Microscope			FEI Titan Krios G3i TEM		
Camera			Gata K3 with BioQuantum GIF		
Magnification			64,000		
Voltage (kV)			300		
Electron exposure (e^{-}/Å)			32 - 36		
Total frames			40		
Defocus range (μm)			-0.8 - -3.0		
Pixel size (Å/pixel)			1.34		
Symmetry imposed			C1 and helical		
Initial particle number			3,928,235		
**Refinement**					
Map resolution (Å)	6.4	4.1	5.8	3.8	4.0
Final particle number	41,701	136,681	19,787	231,875	124,254
Map sharpening b factor	0	0	0	-128	-109

Table S4. Cryo-EM data collection and refinement statistics for the transitional A-C linker.

	Composite map Transitional (EMDB-53987)	A-link & A-microtubule (EMDB-53984)	C-link & C-microtubule (EMDB-53986)	C-link focused (EMDB-53985)
**Data collection and processing**				
Microscope	FEI Titan Krios G3i TEM			
Camera	Gata K3 with BioQuantum GIF			
Magnification	64,000			
Voltage (kV)	300			
Electron exposure (e^{-}/Å)	32 - 36			
Total frames	40			
Defocus range (µm)	-0.8 - -3.0			
Pixel size (Å/pixel)	1.34			
Symmetry imposed	C1 and helical			
Initial particle number	3,928,235			
**Refinement**				
Map resolution (Å)	N/A	3.9	3.8	3.8
Final particle number	N/A	393,606	290,362	104,864
Map sharpening b factor	N/A	-112	-133	-124

Titles for supplemental auxiliary files

Table S5. List of proteins identified in *Tetrahymena* isolated centrioles by MS (replicate 1).

Table S6. List of proteins identified in *Tetrahymena* isolated centrioles by MS (replicate 2).

Table S7. List of proteins identified in *Tetrahymena* isolated centrioles by MS (replicate 3).

Table S8. Measurement and statistics of centriolar modules.

Video S1. Overlay of intracellular component segmentation results with slices through the cryo-tomogram of cryo-FIB-milled lamellae of *Tetrahymena* cell shown in Fig. 1A.

Video S2. Slices through the cryo-tomogram of an isolated *Tetrahymena* centriole shown in Fig. 1F.
